# Mechanical Performance and Artificial Aging Behavior of Reinforced 3D-Printed PLA Structures for Drone Arm Application

**DOI:** 10.3390/polym18080963

**Published:** 2026-04-15

**Authors:** Miloš R. Vasić, Miloš D. Vorkapić, Danica M. Bajić, Snežana B. Vučetić, Marija K. Kovač, Anja Terzić, Biljana Ilić

**Affiliations:** 1 Institute for Testing of Materials, Bulevar Vojvode Mišića 43, 11000 Belgrade, Serbia; anja.terzic@institutims.rs (A.T.); biljana.ilic@institutims.rs (B.I.); 2 Institute of Chemistry, Technology and Metallurgy, National Institute of the Republic of Serbia, University of Belgrade, Njegoševa 12, 11000 Belgrade, Serbia; worcky@nanosys.ihtm.bg.ac.rs; 3 Military Technical Institute, 11030 Belgrade, Serbia; simic_danica@yahoo.com; 4 Faculty of Technology, University of Novi Sad, Bulevar cara Lazara 1, 21000 Novi Sad, Serbia; snezanap@uns.ac.rs (S.B.V.); marija.kovac@uns.ac.rs (M.K.K.)

**Keywords:** additive manufacturing, mesh reinforcement, hydrolytic degradation, PLA, mechanical performance, durability of thermoplastic polymer

## Abstract

This study addresses several key limitations identified in previous research on additively manufactured PLA composites. Unlike most earlier studies that focused primarily on the characterization of as-printed materials, the present work systematically investigates both mechanical and surface behavior before, during, and after artificial aging. In addition, six different printing configurations and reinforcement types (PVC and fiberglass mesh) were analyzed under controlled conditions, enabling a more reliable assessment of their combined influence on composite performance. Printed specimens were artificially aged for 45 and 90 days. The aging protocol combined cyclic changes in moisture, temperature, UV, and IR agents, trying to mimic real exploitation conditions as realistically as possible. The chemical and surface changes during aging were tracked using FTIR spectroscopy, colorimetry, contact angle, and surface free energy measurements. Mechanical performance at 0, 45, and 90 days was evaluated through tensile, three-point bending, and Charpy impact tests, as well as full-scale cantilever loading tests of real printed drone arms. Results show that artificial aging causes measurable chemical and surface modifications, as indicated by changes in the FTIR degradation index and surface wettability. However, these changes do not result in severe mechanical degradation within the investigated aging period. Reinforcement in the form of incorporated PVC and fiberglass mesh significantly affected failure behavior. Specimens printed with higher infill density and thicker infill lines generally exhibit improved mechanical properties. Specimens stiffness and impact resistance were also altered. Results demonstrate that reinforced PLA structures are suitable for lightweight drone applications.

## 1. Introduction

Additive manufacturing (AM) has become one of the most widespread methods for prototyping and producing functional parts due to its simplicity, low cost, and flexibility. Polylactic acid (PLA) is a prevailing type of thermoplastic filament due to its environmental friendliness, biodegradability, and ease of use. In addition, this filament can be used on printers that do not have a heating chamber, since printing is achieved at relatively low temperatures, typically around 190 °C to 220 °C, with reduced warping risk and more flexible printing control, resulting in more precise product dimensional accuracy and printed surface quality [1,2]. For this reason, PLA is frequently utilized in low-stress components, medical equipment, packaging, and prototypes [3,4].

In recent years, extensive research has demonstrated that the mechanical performance of 3D printed polymers is strongly affected by process-induced anisotropy, interlayer adhesion, and raster orientation. These parameters directly affect tensile, flexural, and impact properties [5,6,7,8]. Recent studies report that tensile strength and stiffness are primarily controlled by interlayer bonding quality and that the flexural behavior is mostly affected by the infill density and structural continuity between the layers of the 3D-printed material [9,10].

The quality of 3D-printed structures is directly correlated with the printing technology used and the selected filament properties. Furthermore, the susceptibility to environmental degradation, for example, thermal warping and hydrolysis, is expected to increase over time. This trend is probably a consequence of the PLA’s semi-crystalline nature and relatively low glass transition temperature (55–60 °C). Printed parts commonly have lower tensile and impact strength in the build direction and, over time, become brittle [11,12]. These limitations are the reason for ongoing research aimed at enhancing the mechanical, interfacial, and aging properties of PLA-based printed structures. The solution is to use reinforced filaments to select a more efficient printing strategy or to employ post-processing procedures. Thus, commercially available reinforced filaments can be employed. Alternatively, custom-made filaments must be created, developed, and produced. The reinforcement strategy is identical in both cases. The fillers, fibers, or nanoparticles are introduced before extrusion. Although this technology enhances the strength, stiffness, thermal stability, surface properties, UV resistance, weathering resistance, and adds opacity to the final product, its drawbacks are probably associated with nozzle jamming. Different types of fillers (diatom, CaCO_3_, metal powders), natural fibers (flax, jute, sisal, kenaf, cotton, rice husk), artificial fibers (basalt, graphene), nanofibrillated (cellulose, wood, bamboo), or nanoparticles/wires (BaTiO_3_) have been used as reinforcement materials (RMs). The RM content ranged from 1 to 30 wt.% [13,14].

The most comprehensive review about natural and artificial RM for FDM application can be found in the study by Li et al. [15]. For example, a composite material with an RM (sisal) content ranging from 1 to 5 wt.% showed improved strain-to-failure ratios compared to the reference PLA. In comparison, those with higher sisal content had reduced tensile strength due to the formation of voids [16]. Generally, PLA filaments reinforced with flax, jute, and rice husk show greater stiffness and biodegradability [17]. Depending on both the fiber fraction and the printing orientation, the resulting materials generally exhibit tensile strengths of 28–80 MPa and elastic moduli of 2–7 GPa. However, when RM content exceeds roughly 20 wt.%, most studies [18,19,20,21,22,23,24] report a notable reduction in elongation at break and, in some cases, a decrease in tensile strength. These drawbacks are mainly due to weak fiber–matrix adhesion, insufficient fiber dispersion, and printing-induced defects (voids). Discontinuous-fiber-reinforced PLA composites produced by FDM are not always easy to process. In addition, the effects of printing parameters, such as raster angle, layer height, and infill pattern (density), on mechanical performance are not always consistent [17,25,26]. In practice, careful optimization and adjustment of printing settings, such as layer height, nozzle and bed temperatures, and printing speed, are essential since these factors strongly correlate with the mechanical properties of the finished part [26,27]. Standard mechanical characterization of additively manufactured polymers typically includes tensile (ASTM D638), flexural (ASTM D790/D7264), and impact (ASTM D6110/Charpy or Izod) testing. These methods provide the insight into stiffness, strength, and energy absorption capacity [6], which are crucial for the further applicability of a material.

The production of continuous fiber-reinforced PLA structures is even more demanding that in the case of neat filament, as it requires specialized equipment that combines in situ impregnation and towpreg extrusion [28,29]. For example, an FDM extruder capable of producing continuous fiber-reinforced PLA composites by simultaneously depositing molten polymer and fibers. In accordance with study by Heidari-Rarani et al. [30], the composites showed a 208% increase in tensile modulus and a 36% increase in tensile strength, though the failure strain decreased by 62% compared to the reference PLA. The idea to chemically or physically modify the RM surface, was an interesting approach for improving the compatibility between PLA and RM [19,31]. In addition, post-processing annealing techniques, which involve controlled heating of the printed specimen followed by slow cooling, can notably increase polymer crystallinity and, consequently, enhance the final product’s strength, stiffness, thermal stability, and resistance to creep deformation [32]. Better results were reported when the annealing procedure was updated, which involved immersion of as-printed products in previously micronized alabaster or salt, followed by the predefined annealing temperature regime [33,34].

On the other hand, hybrid reinforcement strategies, including in situ insertion of mesh or fiber layers during printing, have gained increasing attention as they enable localized strengthening without modifying the filament itself. Such approaches have been shown to significantly improve flexural stiffness and crack-arresting behavior, particularly in layered structures.

Another innovative and opposite strategy for enhancing the mechanical and artificial aging properties of PLA-based printed structures is to use a base filament and reinforce the printed part by adding one or more layers of RM during printing. The potential of this concept was first reported in the study by Vorkapić et al. [34]. Although the research focused on biomedical applications, the results have shown that interlayer reinforcement can significantly alter stress distribution and failure behavior. Another example in which monofilament polyester mesh was placed between the printed base PLA layers was also recently reported [35]. Even though the mesh has improved the mechanical (tensile) properties, the problems related to adhesion between the PLA and the mesh were merely stretched out. In addition, the inserted interlayer RM does not have to be made of a polymer or fibrous material. The wire mesh (WM) can also be used [36]. The most systematic review of current WM–polymer composite production technologies is presented in study by Singh et al. [37].

Despite promising early results, several gaps remain in the current state of the art. First, composite characterization (e.g., flexural/tensile strength, impact resistance, color, contact angle, wettability, microhardness) has been limited to as-printed composites. Most studies focus on a single RM and polymer, with varied interlayer positions and printing settings, hindering systematic comparison of RM effects on composite properties. Given PLA’s sensitivity to hydrolysis and photo-oxidation, long-term structural reliability requires aging and artificial aging tests. Second, the impact of natural and accelerated aging, such as thermal exposure, UV irradiation, and humidity cycling, on PLA-reinforced composites remains underexplored. A recent comprehensive study [38] evaluated the effects of combined aging protocols on PLA-brass heat-staked joints in 3D-printed drone arms, revealing decreased pull-out strength but retained joint functionality. Degradation was primarily at the metal–plastic interface. Notably, drone arms with low infill density maintained substantial load-bearing capacity post-aging, challenging assumptions about PLA’s structural suitability.

To address these gaps, this work systematically investigates multiple printing configurations under consistent testing conditions, enabling reliable assessment of structural architecture on composite performance. Mechanical testing is integrated with microstructural characterization and aging analysis for two reinforced PLA composites, elucidating both immediate strengthening mechanisms and long-term degradation and failure processes. Confirmation that composite properties and PLA-RM adhesion remain intact during service is achieved when post-aging characteristics exceed project thresholds. The artificial aging protocol from [38] was employed with exposure duration doubled.

## 2. Materials and Methods

### 2.1. Materials and Samples Preparation

PLA white filament CREALITY CR (PLA+) was used. Its characteristics are given in Table 1. This material was chosen as a standard, as it is more flexible, stronger, and durable than ordinary PLA filament. A black P polyvinyl chloride (PVC) mosquito mesh with 2 × 2 mm openings and adhesive “Knauf” fiberglass (FG) bandage tape with 5 × 5 mm openings were used as reinforcement. The reinforced material was tested in accordance with the European guideline ETAG 004 [39]. The average tensile strength is reported in Table 2.

The initial specimen was coded as C (pure—without mesh), while reinforced compossite specimens were coded as B (with brown PVC mesh) and W (with white FG mesh), respectively. The designation codes and printing combinations are given in Table 3. Three identical testing groups were formed. Three drone arms with printed combination PO3, as well as four tensile, four bending, and four Charpy specimens corresponding to all 6 printing IDs, formed each testing group. The total number of specimens per material in each group was 75. Specimens from the first group were tested immediately after printing. This group was seen as a reference one.

Specimens from the second and third group were artificially aged in the Binder KBWF 240 climatic chamber (BINDER GmbH, Tuttlingen, Germany), which was equipped with 2 illumination cassettes with IC and UV light. The weathering protocol (AP-I) reported in reference [38] was used. In this study, the number of cycles was doubled. A total of 45 cycles were used. Since the duration of one cycle is two days, artificial weathering lasted for 90 days.

**Figure 1:**
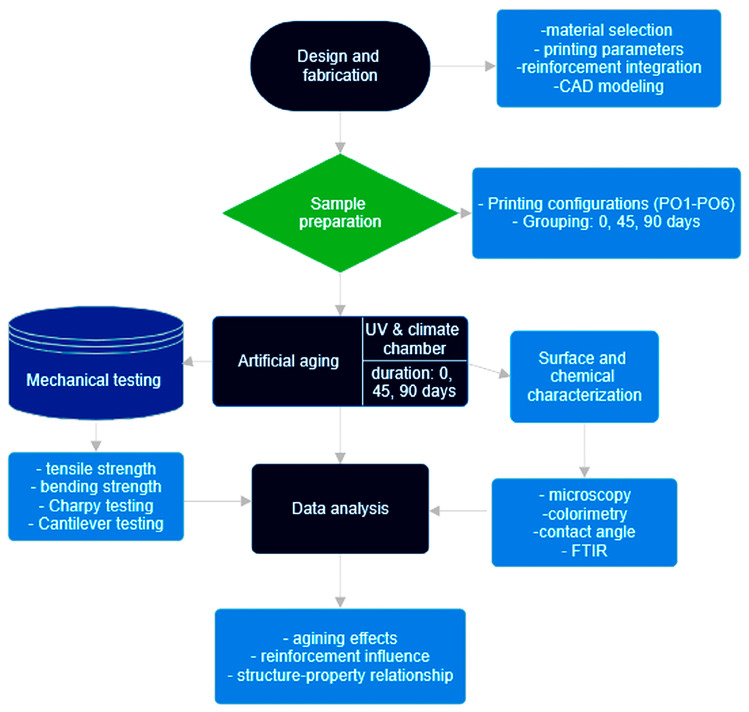
Schematic representation of the experimental workflow.

Specimens from the second group were tested after artificial aging for 45 days, while those from the last group were tested after 90 days of artificial aging. The second group was seen as an intermediate one. The combination PO3 was selected for drone arm printing based on our previous research [38]. In addition, these specimens are larger, and investigation was limited by the weathering chamber’s capacity and by the fact that a large number of specimens (groups two and three) for tensile, bending, and Charpy tests were placed in it simultaneously. A schematic representation of the experimental workflow used in this study is presented in Figure 1.

### 2.2. Mechanical Testing

Specimens for tensile, flexural, and impact strength, with and without reinforcement, were prepared in accordance with the ASTM D638, ASTM D7264, and ASTM D6110 standards. According to ASTM D638, bone-shaped specimens with a total length of 150 mm, a thickness of 3 mm, and an initial distance between the grippers of 106 mm were printed. The dimensions of the other specimens were 80 × 10 × 4 mm and 50 × 10 × 4 mm for the three-point bending test and the impact resistance (Charpy) test, respectively. Each mechanical test was performed on four specimens for each printing configuration and material.

**Figure 2:**
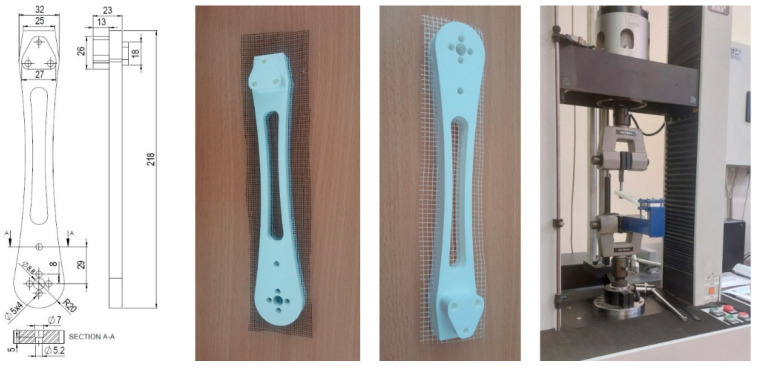
Example of the reinforced drone arms and cantilevered testing.

The Charpy impact pendulum (Zwick, Karl Frank GmbH, Birkenau, Germany) with a maximum impact energy of 7.5 J was used to determine impact resistance. Tensile measurements were conducted on the Shimadzu (Kyoto, Japan) compact table-top universal tester at a constant crosshead speed of 5 mm/min as recommended in ASTM D638. This device’s capacity was 5 kN. The SCHENK TREBEL RM 100 testing device (SCHENCK, Deer Park, NY, USA) was used for three bending tests. The speed of the upper bending tool was 5 mm/min. The distance between the bottom supports (support span) was 50 mm, and the radius of the pressing tool (upper bending tool) was 3 mm. In contrast, the Instron 1122 electromechanical universal testing machine (Instron Worldwide Headquarters, Norwood, MA, USA), equipped with the TRC Pro system for data acquisition and analysis, was used to conduct cantilever bending tests on drone arms. As printed and aged drone arms were placed in a cantilevered position (Figure 2), using a custom-made fixture. An axial force was continuously applied with the speed of 20 mm/min at the arm’s end until failure. The selected mechanical testing methods were chosen to reveal different aspects of structural performance [40,41,42]. Tensile testing provides insight into load-bearing capacity and interlayer adhesion, flexural testing reflects stiffness and resistance to bending, while Charpy impact testing evaluates energy absorption and material toughness under dynamic loading conditions.

### 2.3. Instrumental Analyses

The FTIR analysis, as well as the colorimetric and wettability tests of the material’s surface, were determined for each group only on Charpy specimens (at 0, 45, and 90 days of aging). The recording position was always the same on the upper and lower specimen sides. An FTIR instrument with a diffuse reflectance attachment for non-contact measurements was used. The wavenumber range was 400–4000 cm^−1^, with a resolution of 4 cm^−1^. The FTIR spectrum at each measurement point was calculated as the average of 24 scans. Raw data were analyzed using OPUS (v 9.3; Bruker, Bremen, Germany) and spectrograph software v 1.2.16.

All degradation indices were calculated directly from raw spectral data exported in CSV format. In this way manual peak selection or subjective interpretation of the spectra is avoided. The spectral regions used for degradation index calculation were set before the analysis. The hydroxyl absorption band was quantified within the 3500–3700 cm^−1^ range, while the carbonyl band corresponding to the ester stretching vibration of PLA was evaluated within the 1750–1800 cm^−1^ range. These spectral regions were selected based on vibrational patterns associated with hydrolytic degradation of PLA [43].

To eliminate background and slope effects and ensure that peak intensities and integrated areas reflect only the vibrational absorption of the functional groups of interest, a linear baseline correction was applied before peak quantification. The baseline was obtained by interpolating a straight line between the absorbance values at the boundaries of each chosen spectral window. The corrected absorbance signal was calculated using Equation (1).

(1) $$A\;corrected\left(v\right)=A\;measured\left(v\right)-A\;baseline\left(v\right)$$

Two degradation indexes were calculated: an intensity-based degradation index (DI_peak) and the area-based degradation index (DI_area). The first one was calculated from Equation (2). as the ratio of the maximum corrected hydroxyl peak intensity (IOH) to the maximum corrected carbonyl peak intensity (IC = O). IOH represents the maximum corrected absorbance within the 3500–3700 cm^−1^ spectral range, while IC = O corresponds to the maximum corrected absorbance within the 1750–1800 cm^−1^ band. This index shows the relative increase in hydroxyl groups generated during hydrolytic chain scission compared to the remaining ester carbonyl groups in the PLA backbone.

(2) $${DI}_{peak}=\frac{IOH}{IC=O}$$

The second one was determined from Equation (3). as the ratio of the integrated corrected absorbance area within the hydroxyl region (AOH) to that within the carbonyl region (AC = O). The area under each corrected spectral region was calculated using trapezoidal numerical integration. This index is more convenient when peak broadening occurs during polymer degradation, since more robust results are expected as the integrated absorbance area is used to quantify chemical changes rather than peak intensity.

(3) $${DI}_{area}=\frac{AOH}{AC=O}$$

To enable direct comparison between printing configurations and materials, both degradation indices were normalized relative to the unaged state. Finally, to investigate degradation gradients between exposed and protected regions of the printed structures, the difference between upper and lower surfaces was also calculated. The complete numerical workflow used for spectral processing, baseline correction, peak quantification, and degradation index calculation is provided in the Supplementary Materials.

An objective evaluation and quantification of the material’s color, in accordance with the CIE 1976 standard, in the Lab* color space, was recorded on a handheld spectrophotometer, CM-700D (Konica Minolta, Tokyo, Japan), in SCI recording mode (with the specular component). The aperture size was Φ6 mm (extension of the SAV without a plate), under D65 illumination. The contact angle measurements were recorded on a portable computer-based Advex instrument device (Advex, Brno, Czech Republic).

### 2.4. Printing Technology

The drone arms and specimens for tensile, bending, and Charpy tests were individually drawn in a 3D CAD tool (FreeCAD v 1.1.0). After that, the output file was first converted to STL and then to G-code. Printing was performed on the Creality Ender-6 (Creality, Shenzhen, China) with a 0.4 mm nozzle at a controlled temperature of 24 ± 2 °C and 46% relative humidity. The remaining printing parameters are listed in Table 4.

The insertion technology was the same for both reinforced materials. Practically, the printing process can be temporarily halted at any height of the specimen, allowing the operator to place and secure the reinforcement grid. Subsequently, the printer must be restarted to complete the printing process. In this study, the printer was paused at approximately 50 ± 2% of the specimen’s printed height. The previously cut reinforcement material was placed on top of the printed specimen just after the printer stopped. The printing operator was trained to complete the previous task in the same amount of time. If the duration exceeded the time specified in the time calibration protocol, the specimens were not used, and the process was repeated. A representative example of the as-printed drone arms with reinforcement, along with the product geometry, is shown in Figure 2.

## 3. Results and Discussion

### 3.1. FTIR Analysis

**Figure 3:**
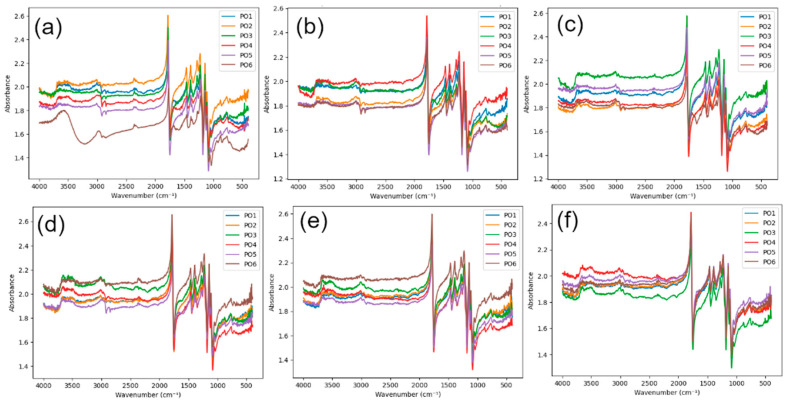
FTIR spectra of C specimens: (**a**) lower surface, 0 days; (**b**) lower surface, 45 days; (**c**) lower surface, 90 days; (**d**) upper surface, 0 days; (**e**) upper surface, 45 days; (**f**) upper surface, 90 days.

**Figure 4:**
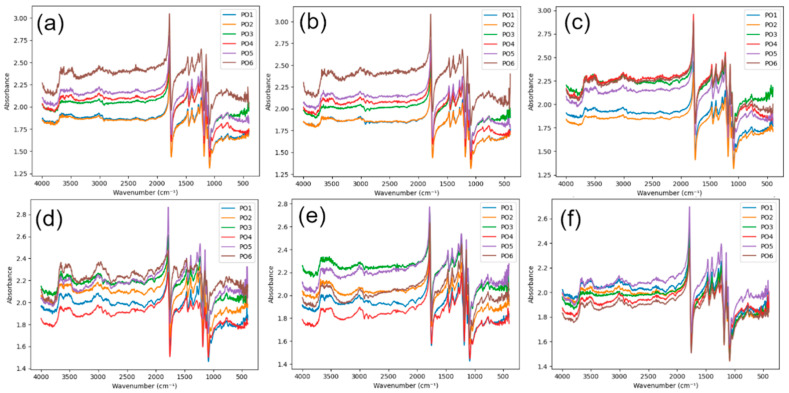
FTIR spectra of B specimens: (**a**) lower surface, 0 days; (**b**) lower surface, 45 days; (**c**) lower surface, 90 days; (**d**) upper surface, 0 days; (**e**) upper surface, 45 days; (**f**) upper surface, 90 days.

**Figure 5:**
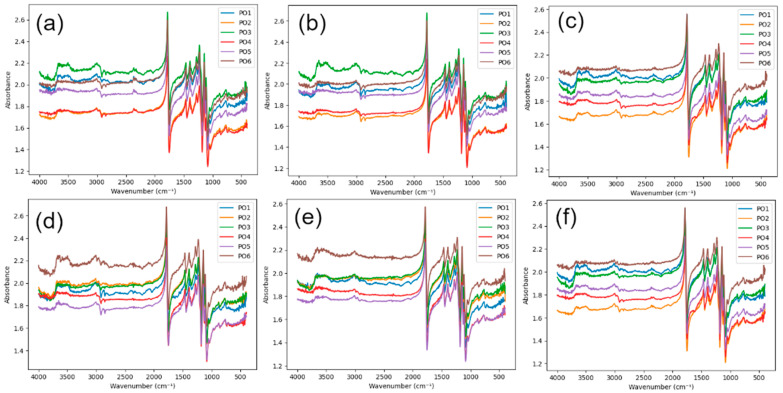
FTIR spectra of W specimens: (**a**) lower surface, 0 days; (**b**) lower surface, 45 days; (**c**) lower surface, 90 days; (**d**) upper surface, 0 days; (**e**) upper surface, 45 days; (**f**) upper surface, 90 days.

FTIR spectra for pure PLA (C), PVC mesh-reinforced PLA (B), and fiberglass-reinforced PLA (W) are presented in Figure 3, Figure 4 and Figure 5. The wavenumbers and absorbance values of all identified FTIR peaks with a threshold of 5% for each printing configuration (PO1–PO6), surface (upper/lower), and aging time (0, 45, and 90 days) are provided in the Supplementary Materials.

All investigated materials exhibited the characteristic absorption bands of PLA. Skeletal, asymmetric C–O–C stretching, CH_3_ bending, C=O ester stretching, aliphatic C-H stretching, and OH group characteristic vibrations were, respectively, found within the 760–880, 1225–1235, 1385–1395, 1750–1785, 2995–3015, and 3500–3650 cm^−1^ spectra regions. A broad absorption band appearing between 3500 and 3650 cm^−1^ is attributed to hydroxyl (OH) groups, which are typically formed as degradation products during hydrolytic cleavage of ester bonds.

The absence of additional peaks in the FTIR spectra of the reinforced materials suggests that no significant chemical changes are detected at the analyzed surface. However, it should be noted that the measurements were performed on the specimen surface, while the reinforcement–matrix interface is located within the bulk of the material. Therefore, the possibility of localized interfacial interactions or formation of new chemical species at the reinforcement interface cannot be excluded. Detecting such effects would require targeted analysis of the interfacial region, which is beyond the scope of the present measurements.

For unaged specimens (0 days), the carbonyl peak appeared sharp and well defined, while the hydroxyl region exhibited relatively low absorbance intensity. This behavior indicates that the ester backbone of PLA remained chemically intact prior to the artificial aging process. After 45 days of artificial aging, moderate spectral changes were observed. A slight broadening of the carbonyl peak and a moderate increase in the intensity of the hydroxyl absorption band were found, probably the result of early hydrolytic PLA degradation, in which water molecules penetrate the polymer matrix and initiate cleavage of ester bonds. Following 90 days of aging, the hydroxyl absorption region appears slightly wider across different printing configurations, while the carbonyl peak shows slight changes in shape and intensity. Nevertheless, the size of these alterations is quite moderate, indicating that the degradation process under the given artificial weathering conditions proceeds slowly. While there are a few differences between the upper and lower surfaces in some configurations, these variations are also quite small, indicating that hydrolytic degradation occurs almost equally on the upper and lower surfaces.

Although qualitative FTIR spectra show what is changing in the material, to truly compare how things break down across different types, print setups, and time, quantitative analysis is necessary. For this purpose, two degradation indices were calculated from the FTIR spectra: the peak intensity-based degradation index (DI_peak) and the peak area-based degradation index (DI_area). The calculated values of these indices for all investigated materials, printing configurations, surfaces, and aging durations are summarized in Table 5 and Table 6. Calculated degradation indices reveal moderate changes during the artificial aging of all the materials considered. Values of DI_peak hold within a fairly limited range close to 0.78 and 0.96, which means that the chemical structure of the PLA matrix, in general, has been maintained to a great extent over the period of aging under investigation.

For pure PLA specimens (C), DI_peak values show only minor variation between the initial state and aged conditions, suggesting that hydrolytic degradation proceeds gradually and does not cause drastic changes in the relative intensities of hydroxyl and carbonyl absorption bands within the investigated aging interval. The reinforced systems exhibit comparable trends.

Since the degradation index values for the B composite (PLA with PVC) are, in most cases, similar to those observed for pure PLA, it can be concluded that the B composites and the as-printed PLA specimens degrade similarly. Registered differences may be associated with variations in moisture transport pathways introduced by the mesh structure within the printed architecture.

The DI_peak changes after artificial aging of composite W (PLA with FG) specimens were moderate. In some configurations, larger variations than those observed with pure PLA are recorded, which can be interpreted as reflecting the hydrophilic character of fiberglass fibers and their possible role in moisture accumulation at the matrix-fiber interface.

The DI_area index spans a wider numeric range than DI_peak, which is why it is considered sensitive to spectral band broadening in the hydroxyl region. Such a phenomenon indicates that hydrolytic degradation may cause distributed chemical changes across the hydroxyl band rather than simply raising the peak maximum intensity. In general, both degradation indices indicate that artificial aging promotes incipient chemical changes in the PLA matrix, though these changes are generally mild.

**Figure 6:**
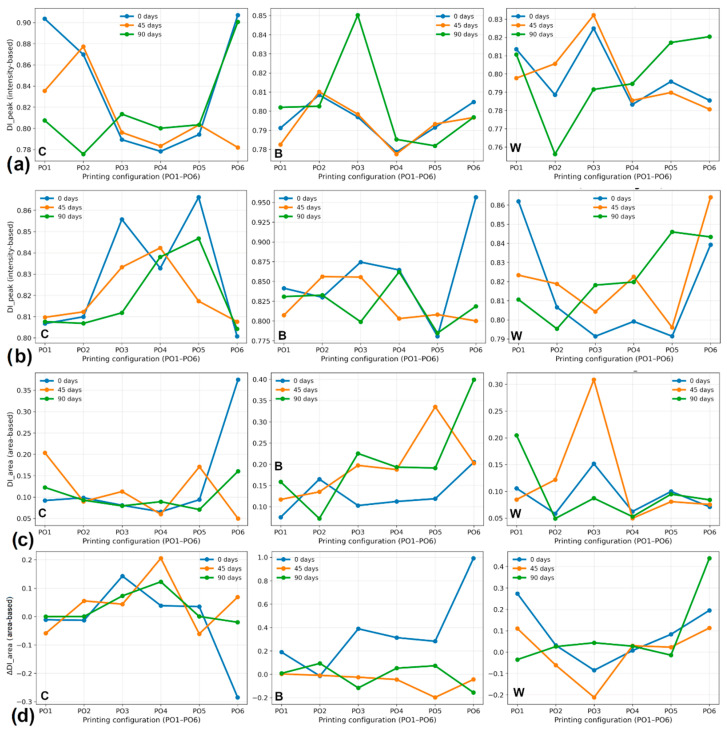
ΔDI Degradation index evolution for pure (C) and composite materials (B, W): (**a**) lower surface; (**b**) upper surface; (**c**) lower surface; (**d**) upper surface.

To better evaluate the evolution of chemical degradation during artificial aging, changes in degradation indices were calculated for three aging intervals: 45–0 days (ΔI), 90–0 days (ΔII), and 90–45 days (ΔIII). The calculated differences are presented in Figure 6. The results indicate that the majority of changes in the degradation index remain relatively small, confirming that chemical aging proceeds gradually over the investigated exposure period. In many configurations, the ΔI values are relatively small, suggesting that only moderate chemical modifications occur during the first 45 days of aging. The ΔII values, representing the overall change between the initial and final aging stages, indicate somewhat larger variations in several configurations. However, these changes remain within a relatively limited numerical range, supporting the conclusion that hydrolytic degradation develops progressively rather than abruptly. The ΔIII values provide additional insight into degradation kinetics between the intermediate and final aging stages. In several configurations, these values remain close to zero, suggesting that the rate of chemical degradation may decrease during the later stages of artificial aging.

The influence of reinforcement type on degradation evolution is also visible in Figure 6. A relatively moderate change in degradation index was registered for B composites. W composites occasionally show somewhat larger variations. This behavior may be associated with differences in moisture transport and local interfacial interactions within the composite structures.

**Figure 7:**
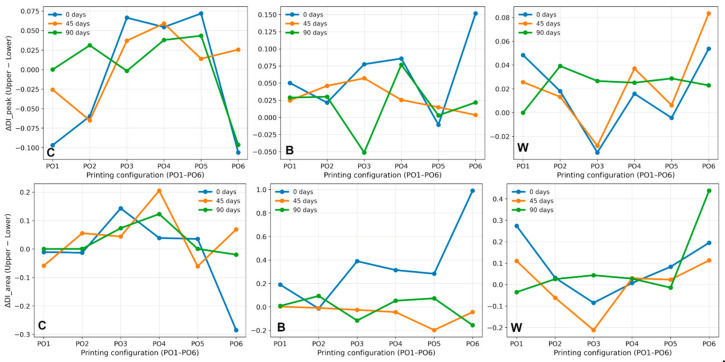
DI (Upper − Lower) evolution during artificial aging.

To investigate possible surface-related degradation gradients, the difference between degradation indices measured on the upper and lower specimen surfaces was calculated (U − D). The resulting values are presented in Figure 7 for both DI_peak and DI_area indices. Based on the calculated upper and lower DI_peak differences, it can be concluded that degradation occurs relatively uniformly across both specimen surfaces under the applied aging conditions. The differences are small and mostly within ±0.10. When the DI-area-based differences are analyzed, more interesting statements about degradation evolution emerge: no strong or systematic degradation gradients were observed between the specimen surfaces. The moderate values of the degradation indices support the previous statement. In addition, the index’s greater sensitivity to distributed spectral changes is evident. Despite the fact that some printing configurations have slightly higher degradation indices on one surface, suggesting local differences in moisture exposure during aging, the summary results indicate that artificial aging affects both surfaces of the printed structures in a relatively comparable manner.

The FTIR analysis showed that hydrolytic degradation is the primary chemical aging mechanism. This is reflected in the gradual broadening and increased intensity of the hydroxyl absorption bands, along with moderate variations in the carbonyl region. While the reinforcement type and printing configuration can, in certain cases, influence the values of the degradation indices, the overall chemical evolution remains relatively moderate during the 90-day artificial aging period investigated. Results suggest that the PLA matrix maintains a relatively stable chemical structure under the applied aging conditions. At the same time, hydrolytic processes gradually modify the molecular structure by cleaving ester bonds and forming hydroxyl end groups.

### 3.2. Colorimetry and Surface Properties

**Figure 8:**
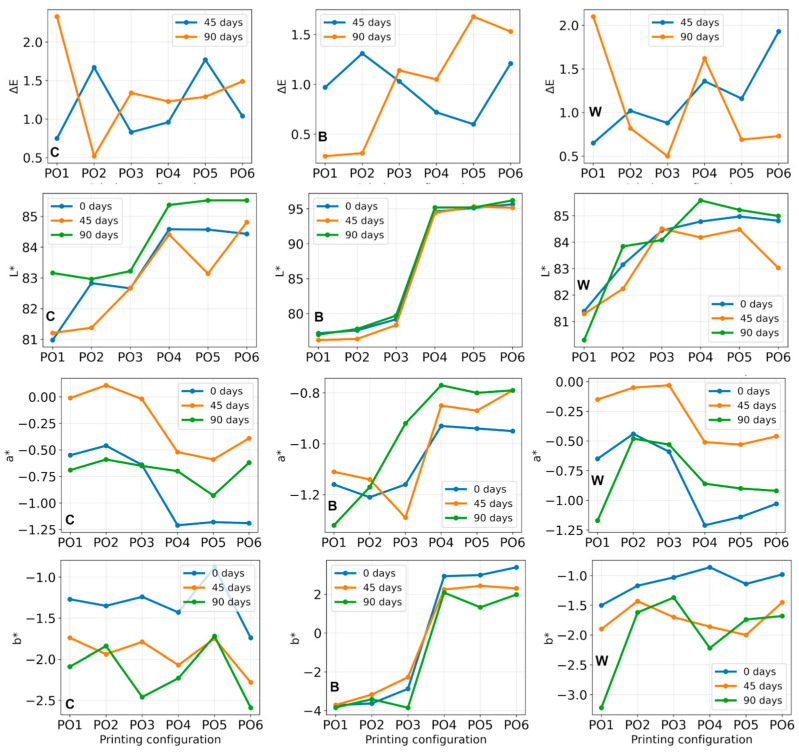
Colorimetric results obtained at 0, 45 and 90 days.

The colorimetric evolution of the investigated materials during artificial weathering is presented in Figure 8 through the ΔE, L*, a*, and b* parameters.

The total color difference (ΔE) value was low for most aged specimens. Its values ranged from approximately 0.60 to 1.93 after 45 days, depending on the material and printing configuration. Somewhat larger variations were observed after 90 days, with ΔE reaching 2.33 in some configurations.

The largest color variation after 90 days was observed in cases of pure PLA (C) for PO1 (ΔE = 2.33), while the remaining configurations generally exhibited values between 0.52 and 1.49. Similar behavior was observed for the reinforced systems. The B composites showed lower color differences after 90 days. Values were mostly below 1.7, indicating that in an optical sense, the surface properties were stable during aging. The W composite exhibited moderate color changes, with ΔE values ranging from 0.50 to 2.10. The highest value was recorded for PO1 after 90 days (ΔE = 2.10). Overall, the ΔE values remained relatively small, indicating that artificial aging resulted in only limited visible color modification of the investigated materials.

The lightness parameter L* exhibited relatively small changes during aging for most specimens. For pure PLA (C), L* values generally increased slightly after 90 days, suggesting a minor brightening of the surface. For example, PO1 increased from 80.98 to 83.16, and PO4 increased from 84.58 to 85.37. The variation in the L* parameter was below the threshold of visual perception.

PVC-reinforced specimens (B) showed very stable L* values throughout the aging period. The variations were typically below ±1 unit, indicating minimal change in brightness. The FG system (W) showed small fluctuations across printing configurations, with overall variations remaining limited. Some specimens showed a slight increase in L* (e.g., PO4), while others showed small decreases (e.g., PO1). These results indicate that artificial weathering did not significantly affect the overall brightness of the investigated materials.

The a* parameter remained negative for most specimens, indicating that the materials retained a slight greenish hue. Small shifts toward less negative values were observed in some configurations after 45 days, followed by a return toward initial values after 90 days. For instance, in the C-PO1 specimen, the change from −0.55 to −0.01 and then to −0.69 was observed, whereas in the W-PO1 specimen, the corresponding change was from −0.65 to −0.15 and then to −1.17. These variations remain relatively small, indicating limited chromatic shifts during aging.

The b* parameter generally shifted toward more negative values after aging, particularly in the pure PLA and fiberglass systems. For example, C-PO6 changed from −1.74 to −2.59, while W-PO1 changed from −1.50 to −3.22. This trend suggests a slight shift toward the blue end of the color space. At the same time, the magnitude of change remained moderate. Overall, the chromatic parameters indicate minor color shifts rather than strong discoloration.

**Figure 9:**
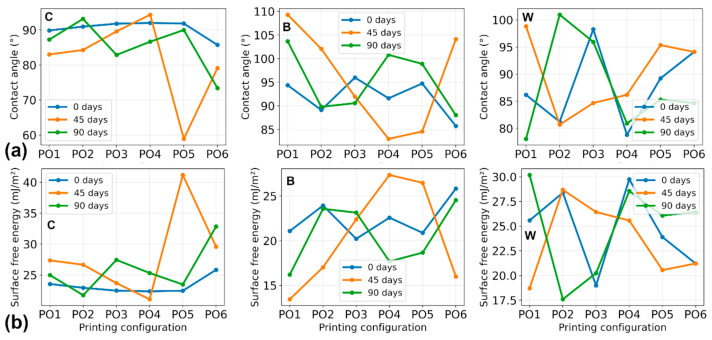
The change in contact angle and surface free energy after 0 45 and 90 days: (**a**) contact angle evolution; (**b**) surface free energy evolution.

The results of the contact angle and calculated surface free energy values are presented in Figure 9. Initial contact angles for most specimens ranged from approximately 80–95°, indicating moderately hydrophobic surfaces typical of PLA-based materials.

From Figure 9, it is evident that during aging, some specimens showed a temporal reduction in contact angle after 45 days, indicating increased surface wettability. For example, the drop from 91.83° to 58.9° was registered in the specimen C-PO5. However, after 90 days (many specimens), the value increased again to 89.99°. The returning value is very close to the initial one. Similar fluctuations were observed for several B and W configurations. Such behavior is expected during the aging of PLA, as the initial decrease in contact angle is associated with surface oxidation and the formation of polar groups that increase wettability, while prolonged exposure can lead to molecular reorientation and partial recovery of hydrophobicity.

B composites often showed higher contact angles, in some cases exceeding 100°. For example, a 109.3° value was registered for B-PO1 (45 days). These results indicate that surface wettability evolves during artificial aging but does not show a uniform monotonic trend. The calculated surface free energy values ranged from approximately 17 to 32 mJ/m^2^ for most specimens. For Pure PLA (C) specimens, several configurations showed an increase after 45 days, followed by stabilization or reduction after 90 days. For example, C-PO1 changed from 23.54 to 27.36 and then to 24.98, while C-PO6 has risen from 25.83 to 29.59 and then to 32.83. B and W composites showed more configuration-dependent variable behavior, which is visualized in Figure 9. This pattern means that some specimens showed an increase in surface energy, while others showed a decrease. Overall, the surface free energy results indicate moderate changes in surface energetic properties during artificial aging.

As moderate changes in the degradation index during aging were calculated, the FTIR data indicated gradual chemical modification of the PLA matrix. The observed variations in wettability and surface energy are consistent with the formation of new functional groups on the polymer surface (caused by degradation), which can influence surface polarity. Despite changes in surface polarity, the magnitude of the colorimetric parameters remained stable, indicating that the chemical modifications found by FTIR did not produce significant optical changes during the investigated aging interval. The combined results indicate that artificial weathering induces moderate chemical and surface property evolution, while the overall visual appearance of the materials remains largely preserved.

Additional microscopy images of specimen surfaces for all configurations, together with colorimetric results, representative color images, and contact angle/surface free energy data, are provided in the Supplementary Materials.

### 3.3. Tensile Test Results

**Figure 10:**
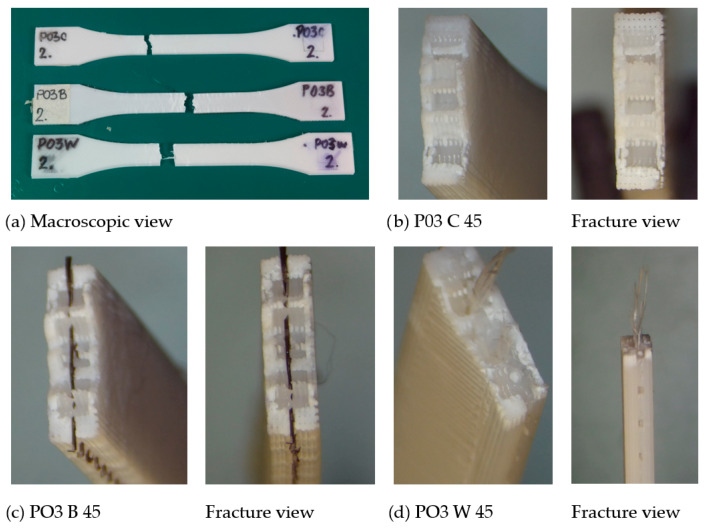
Macroscopic and fracture views of PO3 tensile test specimens after 45 days of artificial aging: (**a**) macroscopic view; (**b**) PO3 C; (**c**) PO3 B; (**d**) PO3 W.

**Figure 11:**
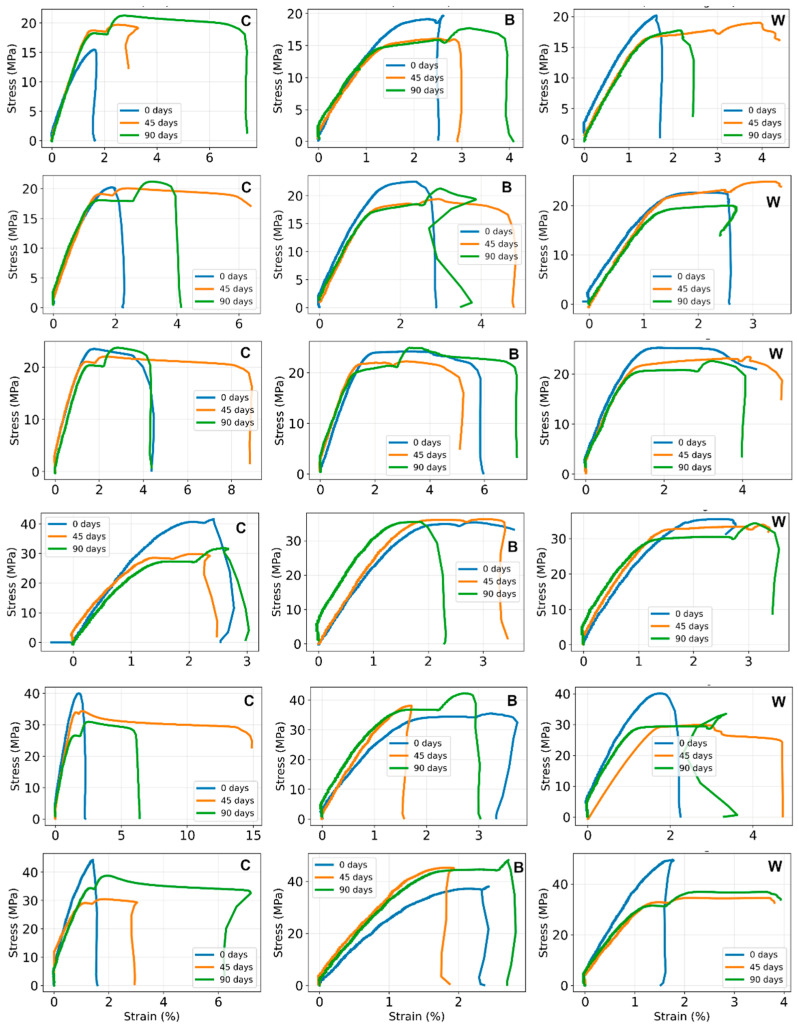
Tensile stress—strain curves.

**Figure 12:**
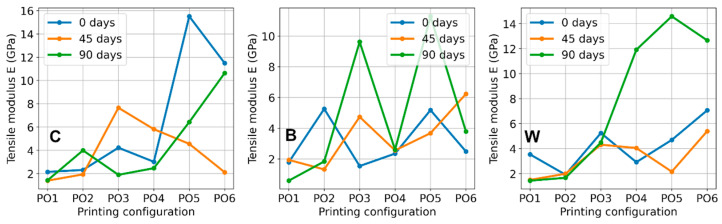
Evolution of tensile modulus during aging.

The representative fracture images of the PO3 configuration after 45 days of artificial aging are presented in Figure 10. Test results are summarized in Table 7 and Figure 11 and Figure 12, with standard deviations of the measured values provided in Supplementary File. This configuration was selected due to its use in the fabrication of drone arm components, providing direct relevance to structural performance. The tensile properties of the investigated materials show a clear dependence on printing configuration, reinforcement type, and artificial weathering duration. The results indicate that the mechanical response does not evolve monotonically with aging, but rather changes in a configuration-dependent manner. The fracture images show visible layer-wise morphology and crack propagation along the printed raster. For C specimens, the fracture surfaces appear relatively uniform, with crack propagation following the layered structure. In contrast, for B and W specimens, the reinforcement mesh is visible within the fracture region.

Despite the presence of reinforcement, the overall fracture appearance remains comparable across all materials and aging conditions, which is consistent with the mechanical results showing convergence of tensile strength values after 45 and 90 days. These observations indicate that artificial aging does not significantly alter the tensile failure mechanism.

A pronounced effect of printing configuration is observed in all three material systems. The as-printed configurations with lower layer height and infill density (PO1–PO3) generally exhibit lower tensile strength than the higher configurations (PO4–PO6). This trend is especially evident in pure PLA (C) and reinforced materials, where the highest tensile stresses are mostly observed in the PO_4_–PO_6_ range. For example, in pure PLA, the tensile strength increases from 15.30 MPa (PO1) to 44.24 MPa (PO6), while in the FG + PLA system, it increases from 19.62 MPa (PO1) to 49.16 MPa (PO6). A similar increase is observed in the PLA + PVC system. These results indicate that the structural parameters introduced during printing strongly influence load-bearing capacity under tensile loading.

The influence of reinforcement on tensile strength depends on both configuration and aging stage. The as-printed reinforced materials often have tensile strengths comparable to or higher than those of pure PLA, particularly in higher printing configurations with larger layer height and infill density. The most pronounced improvement of tensile strength is observed in W composites, where the maximum tensile strength reaches 49.16 MPa for PO6, the highest value in the initial series. Although its behavior is more variable, the strengthening effect was also found in several B composite configurations. In some cases, the reinforced systems do not uniformly exceed pure PLA, indicating that the tensile response is governed not only by the presence of reinforcement but also by the interaction between the reinforcement structure and the printed architecture.

Artificial weathering changes the tensile response, but the effect is not uniform for all material systems. After 45 days, tensile strength generally remains in the same order of magnitude as in the initial state, although the direction of change depends on configuration. Tensile strength is reduced for some specimens. In contrast, for others, comparable or slightly increased values indicate that intermediate aging does not cause catastrophic tensile deterioration but does affect the stability of the tensile response.

After 90 days, the differences between material systems become clearer. For composite B, the highest tensile strength is 48.21 MPa (PO6), while for composite W, it is 36.08 MPa (PO6). In pure PLA, the highest value after 90 days is 33.08 MPa (PO6). Overall, the reinforced materials retain higher tensile resistance than pure PLA after prolonged artificial aging. The strain values also show a strong dependence on aging. After 45 days, a markedly increased strain was observed for some pure PLA and W configurations, such as PO5C (14.01%) and PO3W (5.00%), which represents a more deformable tensile response. Finally, after 90 days, the strain values have returned to more moderate levels, showing that the temporary increase in deformability observed at intermediate aging times was not maintained after prolonged exposure.

The tensile modulus exhibits the greatest variability among the measured tensile parameters. This variability is particularly pronounced in the pre-aged 0-day datasets, where certain configurations demonstrate significantly higher modulus values compared to others. Consequently, the modulus is strongly influenced by both the printing configuration and the quality of the initial linear segment of the tensile curve.

Despite this variability, distinct trends emerge. Elevated modulus values are frequently observed in reinforced systems, especially after 90 days, within printing configurations (PO4–PO6) characterized by higher layer height and infill density. For instance, the W composite attains modulus values of 11.92 GPa (PO4), 14.59 GPa (PO5), and 12.67 GPa (PO6), while the B composite reaches 11.28 GPa (PO5). These findings suggest that prolonged artificial weathering may enhance stiffness in selected configurations, particularly in reinforced structures.

Overall, the tensile results indicate that printing configuration is a primary determinant of tensile performance, as expected according reported studies [41,42]. Reinforcement generally enhances tensile resistance, with this effect becoming more pronounced following extended aging periods. However, these effects vary across configurations, resulting in a non-monotonic degradation pattern due to artificial weathering. Specifically, some specimens exhibit reduced deformability and increased stiffness after 90 days, whereas others maintain properties closer to their initial state. These results imply that the tensile performance of the printed materials is governed by the combined influence of internal architecture, reinforcement type, and structural modifications induced by artificial aging.

### 3.4. Bending Test Results

**Figure 13:**
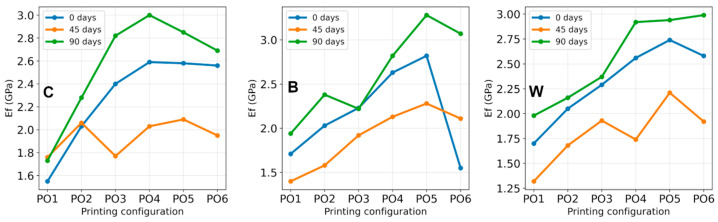
Evolution of E_f_ modulus during aging.

Bending test results are summarized in Table 8 and Figure 13. The results of tests on bending resistance for the examined specimens were registered as force-deflection curves, which were transformed to stress–strain curves. From the obtained curves, the following flexural parameters were determined: F_max_—maximum force, σ_max_—maximum stress, ε_max_—maximum strain, and E_f_—flexural modulus. Standard deviations of the measured values are provided in Supplementary File.

The flexural strength obtained from three-point bending testing shows a systematic dependence on printing configuration, reinforcement type, and artificial aging duration. Irrespective of the materials, all specimens printed with higher layer height and infill density (PO4–PO6) generally exhibit higher maximum flexural stress (σ_max_) than those printed with lower infill density (PO1–PO3), regardless of aging condition. For example, in the unaged state, σ_max_ increases from 46 MPa for PO1 to approximately 72 MPa for PO6 in pure PLA, and similar increases are observed for the reinforced materials.

This behavior is consistent with previous studies [5,40,44,45,46], which show that increased infill density reduces internal porosity and improves stress distribution, thereby enhancing load-bearing capacity and flexural stiffness. During three-point bending tests, the outer fiber region undergoes the highest tensile stress. Specimens fabricated with a higher layer height (0.3 mm) generally exhibit slightly greater σ_max_ values compared to those printed with a 0.1 mm layer height, particularly at elevated infill densities. Minor deviations from this pattern observed in certain configurations are attributed to typical manufacturing variability rather than systematic parameter effects.

The flexural strength and modulus values of the as-printed reinforced specimens are comparable to or slightly higher than those of pure PLA. The FG-reinforced specimens (W) consistently exhibit the highest σ_max_ and E_f_ values, indicating that the reinforcement effectively enhances load transfer between the PLA matrix and the reinforcing structure. Reinforcement located near the specimen’s outer surfaces significantly improves resistance to tensile stresses and delays crack initiation. The B composite also demonstrates increased stiffness across several configurations, although the differences relative to pure PLA vary depending on the printing configuration.

Artificial aging induces a non-monotonic evolution in the flexural properties. Most specimens show reductions in σ_max_ and E_f_ values after 45 days compared to fresh specimens. For instance, in pure PLA, flexural strength decreases from approximately 46–72 MPa (0 days) to 41–54 MPa (45 days), depending on the printing configuration. Concurrently, the strain at maximum stress (ε_max_) increases, in several cases exceeding 10–15%. This combination of reduced strength and stiffness, alongside increased deformability, indicates a temporary softening of the mechanical response, which may be attributed to early stages of hydrolytic degradation or moisture-induced plasticization of the PLA matrix during artificial weathering.

After 90 days of artificial aging, the mechanical response changes markedly. Both σ_max_ and E_f_ increase relative to the 45-day condition, and in many cases, the flexural strength equals or surpasses that of the fresh specimens. For example, pure PLA specimens reach σ_max_ values of approximately 74–78 MPa, whereas B composite (reinforced PLA + PVC) specimens attain values up to 90 MPa. Simultaneously, ε_max_ decreases compared to the 45-day condition, indicating reduced deformability.

This behavior suggests an increase in stiffness accompanied by diminished ductility. Similar trends have been reported in studies on thermally aged PLA [45,46,47,48,49,50], where physical aging and secondary crystallization enhance stiffness and flexural strength by restricting polymer chain mobility.

Based on the three-point bending results, artificial aging appears to induce a two-stage mechanical response in the investigated materials: (1) An intermediate softening stage (45 days), characterized by reduced stiffness and strength with increased deformability. (2) A subsequent stiffening stage (90 days), characterized by increased flexural strength and modulus, accompanied by reduced strain. This behavior aligns with previously reported degradation and physical aging mechanisms in PLA systems [45,46,47,48,49,50].

**Figure 14:**
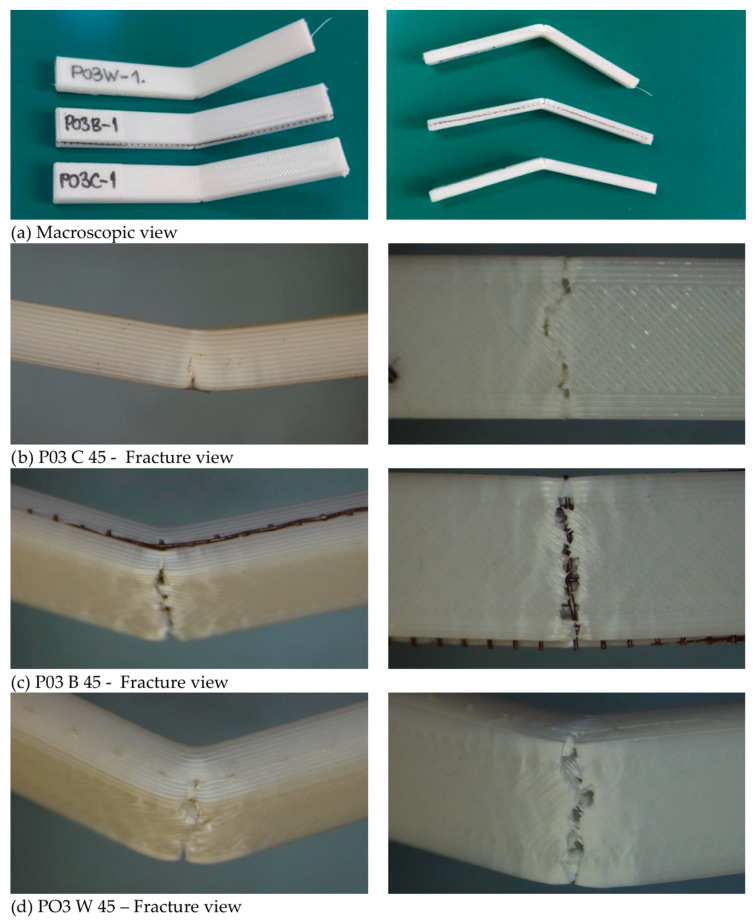
Macroscopic and fracture views of PO3 bending test specimens after 45 days of artificial aging: (**a**) macroscopic view; (**b**) PO3 C; (**c**) PO3 B; (**d**) PO3 W.

The introduction of mesh reinforcement modifies the stress transfer mechanisms during bending. Similar to fiber-reinforced composites, the embedded mesh can act as a load-bearing skeleton, redistributing tensile and compressive stresses across the structure and delaying crack initiation. However, the effectiveness of this mechanism strongly depends on interfacial adhesion between the PLA matrix and the reinforcement. This is why the good mesh-PLA contact must be obtained when inserting mesh between the PLA layers, since this is where load redistribution and crack-bridging/crack-arrest take place, and contribute to enhanced flexural performance [14]. Representative fracture images for the PO3 configuration after bending tests are shown in Figure 14.

The flexural behavior of the materials reflects combined tensile and compressive loading through the specimen thickness and shows a clear dependence on both reinforcement and artificial aging. The fracture surfaces exhibit heterogeneous morphology characteristic of bending-induced failure. For C specimens, fracture propagates through the layered structure, primarily along interlayer regions. In B and W specimens, the reinforcement mesh is visible within the fracture region, indicating its presence along the fracture path. No pronounced differences in fracture morphology are observed between aging conditions. This is consistent with the mechanical results, which show a decrease in strength after 45 days followed by recovery at 90 days, suggesting that the observed changes are related to material response rather than visible structural degradation.

### 3.5. Impact Resistance Test

**Figure 15:**
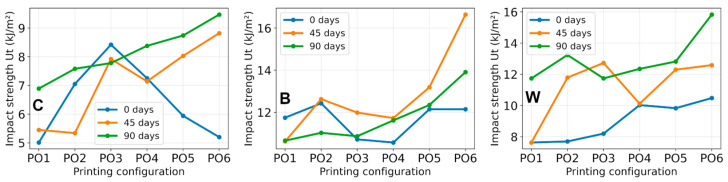
Evolution of impact strength during artificial aging.

The Charpy impact test results are presented in Table 9 and Figure 15. Standard deviations of the measured values are provided in Supplementary File.

Impact results reveal a clear influence of reinforcement type, printing configuration, and artificial weathering on the fracture behavior of the printed specimens.

Across all printing configurations and aging conditions, reinforced materials demonstrate superior impact resistance compared to pure PLA. In the unaged state, pure PLA specimens exhibit ultimate impact energy (U_t_) values of approximately 5–8 kJ/m^2^, whereas reinforced specimens achieve values exceeding 10 kJ/m^2^ in several configurations. The highest impact energies were recorded in reinforced materials, particularly under higher printing configurations. For instance, following artificial aging, B composite specimens reach values up to 15.8 kJ/m^2^, while W composite specimens attain approximately 13–14 kJ/m^2^. In contrast, pure PLA remains below 10 kJ/m^2^ even under the most favorable configurations. This disparity indicates that the reinforcing structures enhance fracture energy by facilitating additional energy dissipation during impact loading.

The impact resistance of the specimens is also correlated with the printing parameters employed. For most materials, impact energy increases progressively from PO1 to PO6, indicating improved fracture resistance in configurations featuring more favorable internal architecture and infill patterns. These factors influence the degree of interlayer fusion and the material’s capacity to resist crack propagation [51,52]. For example, in pure PLA, impact energy rises from approximately 5 kJ/m^2^ in PO1 to 9–10 kJ/m^2^ in PO6, with a similar trend observed in reinforced materials. This pattern suggests that structural parameters introduced during printing significantly affect the specimens’ ability to absorb impact energy prior to fracture.

Artificial aging does not consistently diminish impact resistance. In several configurations, impact energy measured after aging remains comparable to or exceeds values recorded in the unaged condition. For example, in the pure PLA PO6 configuration, impact energy increases from approximately 5.2 kJ/m^2^ in the unaged state to about 9.5 kJ/m^2^ post-aging. Similar behavior is observed in reinforced materials, where the highest impact energies are often recorded following prolonged aging. These findings indicate that the investigated printed structures retain their impact resistance throughout artificial aging. Specimens labeled 5 and 6 consistently exhibit the highest values, suggesting that their printing conditions optimize material properties, potentially due to enhanced interaction between the polymer filament and the incorporated white mesh [53]. The observed increase in impact resistance is characteristic of annealing relaxation in 3D-printed polymers, attributed to reduced porosity and voids, improved interlayer bonding, and decreased residual stresses within the material [18,32].

Printing configuration also plays a critical role in determining fracture behavior, as the impact response of 3D-printed PLA structures is strongly governed by their inherent brittleness and the presence of internal defects such as voids and weak interlayer interfaces. As a result, energy absorption capacity is generally limited compared to conventionally processed polymers. The incorporation of mesh reinforcement significantly alters the impact response by introducing additional energy dissipation mechanisms. During impact loading, the mesh can act as a crack-bridging element, increasing the energy required for crack propagation and thus improving impact resistance [6,9]. It is well established that FDM-printed PLA exhibits relatively low impact toughness due to its brittle nature and the presence of interlayer defects and internal voids [6].

**Figure 16:**
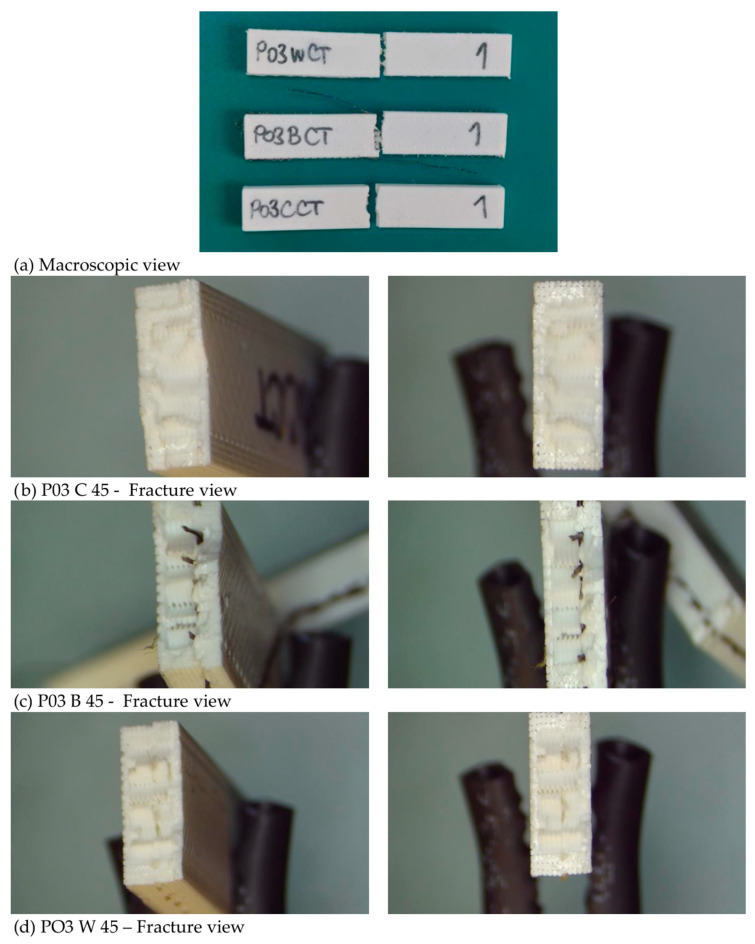
Macroscopic and fracture views of PO3 Charpy test specimens after 45 days of artificial aging: (**a**) macroscopic view; (**b**) PO3 C; (**c**) PO3 B; (**d**) PO3 W.

However, the effectiveness of reinforcement under impact loading is highly dependent on interfacial bonding and mesh positioning. Poor adhesion or incomplete embedding can reduce the reinforcing effect and even act as a defect, which is consistent with observations reported in recent studies on hybrid additively manufactured composites [7]. The impact resistance of the investigated materials is strongly influenced by reinforcement, with composite systems showing significantly higher toughness compared to pure PLA. Fracture images of PO3 specimens after Charpy impact testing are presented in Figure 16.

These images show irregular fracture surfaces typical of dynamic loading conditions. For C specimens, the fracture appears relatively uniform, while in B and W specimens, the fracture surfaces appear more heterogeneous, with the mesh visible within the fracture region. Although no distinct or consistent fracture features can be identified, the increased heterogeneity of fracture surfaces in reinforced specimens is in agreement with the higher impact energy absorption observed in mechanical results. Overall, the results confirm that reinforcement significantly improves impact resistance, while printing configuration governs the extent of energy absorption in additively manufactured PLA structures. Also, these images confirmed that the mesh reinforcements are properly embedded in PLA, without detachment from PLA layers.

### 3.6. Correlation of Mechanical Properties Under Artificial Aging

The combined tensile, flexural, and impact test results demonstrate that the mechanical behavior of the investigated printed materials is primarily influenced by three key factors: printing configuration, reinforcement type, and duration of artificial weathering. Although each test evaluates a distinct loading mode, several consistent trends emerge.

A strong correlation was established between layer height, infill density, and mechanical properties. Superior mechanical performance across all tests is observed in specimens printed with higher layer heights and greater infill densities. This trend is particularly pronounced in tensile and bending tests, where configurations PO4–PO6 generally exhibit higher strength and stiffness compared to PO1–PO3. A similar pattern is evident in the Charpy impact results, with toughness frequently increasing towards PO5–PO6, indicating that the internal architecture introduced during printing significantly influences load transfer, stress distribution, and fracture resistance. Thus, mechanical behavior is governed not only by material composition but also by the structural arrangement within the printed object.

Reinforced materials consistently outperform pure PLA across most mechanical tests, although the extent of improvement varies depending on the loading type. Certain reinforced specimens demonstrated higher tensile strength than pure PLA, particularly after extended aging, albeit with configuration-dependent differences. Under bending loads, the reinforcement effect is more uniform; most composite specimens (B and W) exhibit greater flexural strength and modulus than as-printed PLA, a trend that becomes more pronounced after 90 days. This suggests that reinforcement effectively enhances performance under combined tensile and compressive stresses through the specimen thickness. The most significant reinforcement effect is observed in Charpy impact testing, where B and W composites display substantially higher impact toughness than pure PLA. The patterned hat reinforcement notably improves energy absorption capacity under dynamic fracture conditions. Overall, reinforcement exerts the greatest influence on impact resistance, a marked effect on flexural response, and a more variable impact on tensile behavior depending on configuration.

Mechanical performance did not exhibit a monotonic decline during aging; instead, a two-stage response was identified. The first stage, lasting 45 days, saw reductions in stiffness and strength in several specimens, particularly under bending, accompanied by increased strain, indicative of a temporarily softer, more deformable mechanical response. The second stage, also spanning 45 days, showed recovery or improvement in most properties relative to the 45-day condition. This recovery is especially notable in flexural modulus and certain tensile modulus values, while impact resistance was generally maintained or enhanced. Therefore, prolonged artificial aging does not induce catastrophic mechanical degradation in the materials studied.

The integrated analysis confirms compatibility among all mechanical tests. Tensile testing is most sensitive to structural discontinuities aligned with the loading direction, resulting in greater variability between configurations. Bending testing reflects combined tensile and compressive behavior through the specimen thickness and is clearly sensitive to both reinforcement and weathering effects. Charpy impact testing assesses fracture energy absorption and reveals the most pronounced benefits of reinforcement. Collectively, these findings indicate that reinforced printed structures maintain mechanical integrity better than pure PLA under artificial aging, particularly under bending and impact loading conditions.

Considering all three tests, the highest overall mechanical performance is achieved in the printing configurations PO5 and PO6, combined with added reinforcements. Pure PLA exhibited the lowest mechanical properties. Composite systems demonstrated enhanced resistance to both static and dynamic loading following artificial aging. Consequently, the mechanical results consistently highlight printing architecture and reinforcement type as the primary determinants of performance, while artificial aging modifies but does not supersede these structural effects.

### 3.7. Correlation Between Chemical, Surface and Mechanical Changes

To achieve a comprehensive understanding of material behavior during artificial aging, the mechanical results were correlated with chemical changes identified by FTIR spectroscopy and surface modifications detected through colorimetry, contact angle measurements, and surface free energy analysis. Although these techniques examine different aspects of material behavior, several consistent relationships between chemical, surface, and mechanical evolution were observed.

FTIR analysis revealed measurable chemical changes in the PLA matrix during artificial aging, as indicated by variations in the degradation indices (DI_peak and DI_area). These changes reflect alterations in the relative contributions of hydroxyl and carbonyl absorption bands associated with hydrolytic processes in PLA. The FTIR data processing workflow is presented in the Supplementary Material as Figure S1, while the complete numerical workflow, including structured datasets and degradation index calculations provided as Excel files, is also available. Despite these chemical modifications, mechanical results indicate that the materials do not undergo simple monotonic degradation. Instead, mechanical behavior evolves in a configuration-dependent manner.

At the intermediate aging stage, several specimens exhibited increased strain and reduced stiffness. This behavior aligns with partial chain scission and temporary softening of the polymer structure, which can enhance deformability without necessarily causing immediate strength loss. By the end of the aging period, several specimens demonstrated increased stiffness and flexural resistance compared to those at 45 days. This suggests that additional structural rearrangements occur during prolonged artificial aging, potentially including physical aging or changes in polymer chain packing.

Therefore, FTIR results confirm that chemical modifications occur during artificial aging, while mechanical results demonstrate that these chemical processes do not directly translate into immediate mechanical degradation.

Surface characterization further supports the FTIR findings. Overall color changes (ΔE) were relatively small for most specimens, remaining within moderate ranges. These results indicate that artificial aging conditions induce limited visible surface degradation. Concurrently, measurable changes were observed in contact angle and surface free energy. In several cases, the contact angle decreased at intermediate aging times, indicating increased surface wettability. This behavior is consistent with the formation of polar surface groups, as suggested by the increase in hydroxyl-related FTIR signals. The Supplementary Material includes microscopy images, colorimetric data, and surface-related analyses supporting the observed surface phenomena during ageing.

Surface free energy values also exhibited moderate variations during aging, reflecting changes in surface polarity and chemistry. These modifications align with the hydrolytic processes detected by FTIR spectroscopy. The surface measurements further support the conclusion that artificial aging primarily affects surface chemistry, while the bulk structure remains sufficiently intact to preserve mechanical performance.

Impact test results reinforce the interpretation that reinforcement and structural configuration significantly influence mechanical performance. Composite (reinforced) materials exhibited substantially higher Charpy impact toughness than as-printed PLA under all aging conditions, indicating that reinforcement effectively contributes to energy absorption during fracture, even when surface chemistry changes occur during artificial aging. The enhanced toughness of reinforced materials suggests that reinforcement structures help identify crack propagation and redistribute stresses within the printed structure. This behavior explains why reinforced specimens maintain high impact toughness despite surface modifications detected by colorimetric and wettability measurements.

To provide a comprehensive overview of material aging, chemical, surface, and mechanical results were cross-validated. Artificial aging induces measurable chemical changes in the PLA matrix, as confirmed by FTIR degradation indices. These chemical modifications coincide with moderate changes in surface properties, including color, wettability, and surface free energy. Simultaneously, mechanical results demonstrate that the structural integrity of the printed materials is largely preserved. Mechanical behavior remains strongly influenced by printing configuration and reinforcement type. Although artificial weathering primarily causes changes in surface chemistry and molecular structure, the degree of chemical degradation observed during aging remains secondary.

In contrast, the overall mechanical performance of the printed structures remains stable, particularly in reinforced configurations. Ultimately, the combined results indicate that artificial weathering produces detectable chemical and surface modifications in the investigated PLA-based materials, while mechanical behavior remains predominantly governed by structural factors such as printing configuration and reinforcement type. Reinforced materials, especially those with higher printing configurations, maintain superior mechanical performance despite chemical changes detected by FTIR spectroscopy.

The observed changes in surface chemistry and wettability can be attributed to the degradation pathways of PLA under environmental exposure. Moisture and elevated temperature initiate hydrolytic degradation of ester bonds within the polymer backbone, leading to progressive chain scission and reduction in molecular weight. This process gradually increases material brittleness due to reduced chain entanglement and diminished capacity to dissipate mechanical energy. Concurrently, UV radiation promotes surface oxidation reactions that generate oxygen-containing functional groups, detectable through changes in FTIR spectra and surface energy parameters. These combined mechanisms explain the detectable surface and chemical modifications observed after artificial aging, despite the relative stability of bulk mechanical properties within the investigated exposure period.

### 3.8. Cantlievered Drone Arm Testing Results

**Figure 17:**
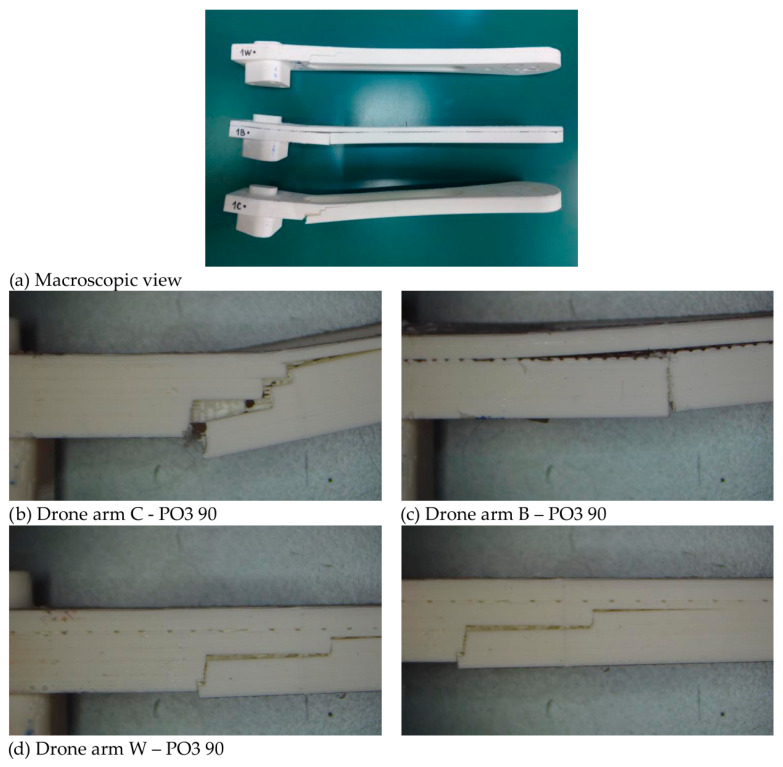
Representative images of drone arm specimens after cantilever bending tests (PO3 configuration, 90 days of artificial aging): (**a**) macroscopic view of tested specimens; (**b**–**d**) detailed views of fracture regions for pure PLA (C), PVC-reinforced (B), and FG-reinforced (W) specimens.

**Figure 18:**
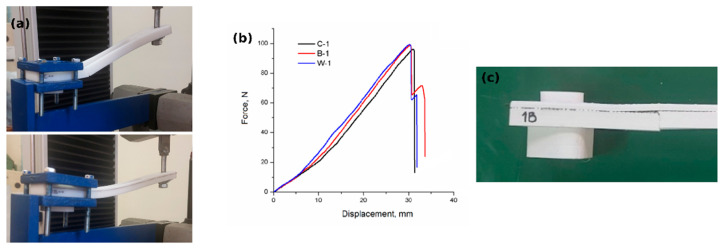
Cantilever bending test: (**a**) drone arm; (**b**) load–displacement curves; (**c**) representative fracture appearance after testing.

To evaluate the structural applicability of the investigated materials in a real component, drone arm specimens were tested under cantilever loading conditions. The tests were performed on fresh specimens and on specimens subjected to artificial aging for 45 and 90 days. This loading configuration simulates the bending stresses that occur in drone arms during operation. Results are summarized in Table 10. Representative microscopy images of the PO3 drone arm specimens are presented in Figure 17. The force-displacement (deflection) curve for specimens aged 90 days was shown as an example in Figure 18.

These images, including side views, clearly show the position of the reinforcement mesh within the structure, located between printed layers and following the geometry of the specimen. The fracture appearance indicates that crack propagation is influenced by the presence of the mesh, and in several regions the crack path appears to be locally interrupted or limited in areas above the reinforcement. These observations are consistent with the mechanical results, which show preserved load-bearing capacity after artificial aging, indicating that the structural role of the reinforcement is maintained despite aging effects.

The as-printed W composite had the highest load (116 N) and the lowest deflection (34.0 mm), indicating the highest bending stiffness among the tested materials. The pure PLA specimen (C) reaches a maximum load of 104 N with a deflection of 48.5 mm, while the B composite reaches 108.5 N with the largest deflection (49.6 mm).

From the load-deflection curve shown in Figure 18, it can be seen that the composite material’s (B, W) stiffness is higher than the corresponding as-printed PLA one. The W composite increased bending rigidity, resulting in lower deformation under load. In contrast, the B composite shows slightly higher deflection, indicating a more compliant response while maintaining comparable load-bearing capacity.

After 45 days, a reduction in the maximum load relative to as-printed drone arms was found for all tested materials. The peak ranged between 83.5 N and 90.5 N, indicating a moderate decrease in cantilever load-bearing capacity. The determined deflection at the peak load position was also reduced compared to the as-printed state. Even though these findings can indicate somewhat stiffer behavior, the shape of the load–deflection curves remained similar to the initial as-printed state, suggesting that the overall failure mechanism was not significantly altered during the first aging stage. These results indicate that artificial weathering initially reduces the load-bearing capability of the printed structures, although the specimens still maintain stable structural behavior.

The specimens artificially aged for 90 days show comparable cantilever load-bearing capacity across all materials. All three specimens exhibit similar maximum forces before failure or sudden load drop, with comparable bending resistance.

The curve obtained for specimen C-1 (Figure 17) shows moderate stiffness followed by a sharp drop in load after reaching the peak force. Such behavior indicates a predominantly brittle fracture mode under cantilever loading. The relatively smaller area under the load–displacement curve suggests slightly lower energy absorption compared to the reinforced specimens.

Specimen B-1 (PLA reinforced with black mesh) reaches a slightly higher peak force and exhibits a more gradual load reduction after the maximum value is reached. The observed pattern of load decrease, progressive rather than a sharp drop, is in line with the observed local interlayer separation or delamination within the printed structure (Figure 17a,c). This behavior indicates a more gradual damage development and suggests that the mesh reinforcement may contribute to partial load redistribution after crack initiation.

The W-1 specimen (PLA reinforced with FG mesh) shows the highest stiffness among the tested specimens and reaches a high peak force followed by a sudden load drop. This behavior indicates that reinforcement increases bending stiffness, but failure still occurs in a relatively brittle manner once the critical stress is reached.

The cantilever tests revealed that the drone arms remained structurally stable under artificial aging conditions. Although a temporary reduction in load capacity is observed after 45 days, the load-bearing capability partially recovers after prolonged aging.

The presence of reinforcement influences primarily the deformation behavior and failure mechanism rather than the maximum load itself. Reinforced specimens exhibit higher stiffness and modified post-peak behavior, while pure PLA tends to fail in a more brittle manner. This mechanism of failure at the cantilever bending test is due to the aging conditions that have probably caused polymer relaxation/crystallization and the embrittlement of the PLA material [54,55].

Obtained results are in line with the trends observed in the standardized mechanical tests. Reinforcement improves stiffness and impact resistance. Simultaneously, the overall structural integrity of the printed component remains preserved after artificial aging. The cantilever test, therefore, confirms the practical applicability of the investigated mesh-reinforced printed structures under realistic loading conditions.

## 4. Conclusions

In this research, the influence of printing parameters, reinforcement type, and artificial aging on the mechanical and surface behavior of additively manufactured PLA-based structures intended for drone arm applications was investigated. The results show that the mechanical performance of the printed specimens strongly depends on the internal printing configuration. The main results and conclusions are summarized below:

- Configurations with higher layer height and infill density (PO4–PO6) generally exhibit improved tensile, flexural, and impact strength compared to lower configurations (PO1–PO3), confirming the important role of structural architecture in load transfer within the printed material.
- Reinforcement also significantly influences mechanical behavior. Composites B and W exhibit improved mechanical performance compared with as-printed PLA specimens, particularly under bending and impact loading. The reinforced specimens demonstrate higher stiffness and improved energy absorption, while the presence of mesh reinforcement modifies the post-peak failure behavior.
- A detailed analysis of mechanical results shows that in lower configurations (PO1–PO2), tensile strength remains limited, ranging from 15.30 MPa to 19.68 MPa in PO1 and up to 21.85 MPa in PO2, with no consistent improvement due to reinforcement. However, a pronounced effect is observed in impact resistance, where reinforced specimens reach up to 11.75 kJ/m^2^ (PO1) and 12.43 kJ/m^2^ (PO2), compared to 5.01–7.05 kJ/m^2^ for pure PLA. In intermediate configurations, tensile strength in PO3 remains comparable across all materials (21.12–21.84 MPa), while in PO4 it increases significantly, reaching 41.59 MPa in pure PLA, with slightly lower values observed for reinforced systems.
- In higher configurations (PO5–PO6), tensile strength and stiffness further increase, with maximum values reaching approximately 49.16 MPa in the fiberglass-reinforced system. Reinforcement effects in this range become more dependent on configuration and aging condition, but overall trends confirm improved structural performance at higher infill densities.
- FTIR analysis indicated changes in surface properties, while contact angle measurements confirmed these findings. However, these chemical and surface changes do not lead to severe mechanical deterioration within the investigated aging period (90 days). Mechanical performance remains largely controlled by printing configuration and reinforcement type.
- FTIR-derived degradation indices remained within a narrow range (DI_peak ≈ 0.78–0.96), with differences between the surfaces below ±0.10, confirming moderate chemical deterioration over 90 days. The DI_area parameter exhibited broader variation but it still did not indicate severe degradation kinetics.
- The cantilever testing of the drone arm specimens confirms the practical applicability of the investigated structures. In the PO3 configuration, reinforced specimens exhibit higher load-bearing capacity compared to pure PLA, with the W specimen reaching approximately 116 N, followed by the B configuration, while the C specimen shows the lowest load values. A temporary reduction in load-bearing capacity is observed after intermediate aging (45 days), followed by stabilization after 90 days, indicating that structural performance is preserved despite environmental exposure.

The reported numerical values correspond to representative results from the analyzed datasets and may refer to different aging stages depending on the configuration and material system.

The obtained results are in agreement with recent studies on additively manufactured PLA composites, which indicate that properly designed reinforcement strategies can reduce the negative effects of environmental exposure while preserving mechanical functionality over time. The results confirm that properly designed reinforced PLA structures can retain sufficient mechanical integrity even after prolonged environmental exposure, supporting their use as lightweight and structurally reliable components in drone systems. These findings contribute to the broader understanding of durability in additively manufactured polymer composites and open new possibilities for their application in lightweight engineering structures operating under real environmental conditions.

## Figures and Tables

**Table 1:** Declared PLA filament characteristic.

Parameter	Property
Diameter	1.75 ± 0.02 mm
Tensile strength	≥60 MPa
Flexibility	≥60 MPa
Density	1.25 ± 0.05 g/m^3^
Elongation at break	≥3%
Water absorption	0.5%
Nozzle temperature	190–205 °C (recommended 200 °C)
Plate temperature	60 °C
Spool weight	1 kg
Filament code	10,100
Manufacturer	Creality Shenzhen, Shenzhen, China

**Table 2:** Characteristics of the PVC and FG reinforced materials.

Reinforced Material	Average Tensile Strength (N/mm^2^)	Standard Deviation
PVC	11.85	0.92
FG	13.18	1.99

**Table 3:** Designation codes.

A. Material Classification						
Code ID	Composite		Reinforcement		Color	
C	Pure PLA		-		White	
B	PLA + PVC		PVC mesh		Brown	
W	PLA + FG		FG mesh		White	
B. Printing combinations						
Combination ID	P01	P02	P03	P04	P05	P06
Layer height (mm)	0.1	0.2	0.3	0.1	0.2	0.3
Infill density (8%)	50	50	50	90	90	90

**Table 4:** Printing parameters.

Parameter	Value	Parameter Type
Infill Pattern	Lines	Fixed
Infill Line direction	+45°/−45°	Fixed
Wall line count	3	Fixed
Top layers	2	Fixed
Bottom layers	2	Fixed
Print speed	60 mm/s	Fixed
Nozzle temperature	210 °C	Fixed
Build plate temperature	60 °C	Fixed
Layer height (mm)	0.1/0.2/0.3	Variable
Infill density (%)	50/90	Variable

**Table 5:** Degradation index for upper surface.

	DI_Peak (C)			DI_Peak (B)			DI_Peak (W)			DI_Area (C)			DI_Area (B)			DI_Area (W)		
	0	45	90	0	45	90	0	45	90	0	45	90	0	45	90	0	45	90
PO1	0.81	0.81	0.81	0.84	0.81	0.83	0.86	0.82	0.81	0.08	0.14	0.12	0.26	0.19	0.17	0.38	0.19	0.17
PO2	0.81	0.81	0.81	0.83	0.86	0.83	0.81	0.82	0.80	0.09	0.15	0.09	0.15	0.13	0.17	0.09	0.06	0.07
PO3	0.86	0.83	0.81	0.87	0.86	0.80	0.79	0.80	0.82	0.22	0.16	0.15	0.49	0.17	0.11	0.07	0.10	0.13
PO4	0.83	0.84	0.84	0.86	0.80	0.86	0.80	0.82	0.82	0.10	0.26	0.21	0.43	0.14	0.25	0.07	0.08	0.08
PO5	0.87	0.82	0.85	0.78	0.81	0.78	0.79	0.80	0.85	0.13	0.11	0.07	0.40	0.14	0.26	0.18	0.10	0.08
PO6	0.80	0.80	0.80	0.96	0.80	0.82	0.84	0.86	0.84	0.09	0.12	0.14	1.20	0.16	0.24	0.27	0.19	0.52

**Table 6:** Degradation index for lower surface.

	DI_Peak (C)			DI_Peak (B)			DI_Peak (W)			DI_Area (C)			DI_Area (B)			DI_Area (W)		
	0	45	90	0	45	90	0	45	90	0	45	90	0	45	90	0	45	90
PO1	0.90	0.84	0.81	0.79	0.78	0.80	0.81	0.80	0.81	0.09	0.20	0.12	0.08	0.12	0.16	0.11	0.08	0.20
PO2	0.87	0.88	0.78	0.81	0.81	0.80	0.79	0.81	0.76	0.10	0.09	0.09	0.16	0.13	0.07	0.06	0.12	0.05
PO3	0.79	0.80	0.81	0.80	0.80	0.85	0.82	0.83	0.79	0.08	0.11	0.08	0.10	0.20	0.23	0.15	0.31	0.09
PO4	0.78	0.78	0.80	0.78	0.78	0.79	0.78	0.79	0.79	0.07	0.06	0.09	0.11	0.19	0.19	0.06	0.05	0.05
PO5	0.79	0.80	0.80	0.79	0.79	0.78	0.80	0.79	0.82	0.09	0.17	0.07	0.12	0.34	0.19	0.10	0.08	0.09
PO6	0.91	0.78	0.90	0.80	0.80	0.80	0.79	0.78	0.82	0.37	0.05	0.16	0.20	0.20	0.40	0.07	0.08	0.08

**Table 7:** Tensile test results.

0 Days	σ_max_ (MPa)			ε_max_ (%)			E_f_ (GPa)		
	C	B	W	C	B	W	C	B	W
PO1	15.30	19.68	19.62	1.78	2.66	1.80	2.15	1.79	3.54
PO2	19.98	21.85	20.74	2.10	2.70	2.75	2.32	5.26	1.92
PO3	21.83	21.12	21.84	3.78	5.68	4.12	4.23	1.54	5.25
PO4	41.59	35.25	33.94	2.44	2.94	2.78	3.02	2.35	2.92
PO5	39.34	34.71	39.58	1.91	3.66	2.14	15.51	5.18	4.70
PO6	44.24	38.01	49.16	1.61	2.47	1.91	11.49	2.49	7.07
45 Days	σ_max_ (MPa)			ε_max_ (%)			E_f_ (GPa)		
	C	B	W	C	B	W	C	B	W
PO1	18.88	15.95	19.02	3.23	2.94	3.97	1.40	1.94	1.50
PO2	18.09	18.01	23.99	6.21	4.26	3.46	1.94	1.32	1.99
PO3	20.16	21.10	20.38	8.48	4.67	5.00	7.66	4.74	4.32
PO4	29.17	35.97	33.82	2.50	3.29	3.37	5.82	2.56	4.05
PO5	28.82	38.04	24.42	14.01	1.76	4.72	4.56	3.68	2.16
PO6	29.35	44.94	34.53	3.53	2.06	3.84	2.12	6.23	5.40
90 Days	σ_max_ (MPa)			ε_max_ (%)			E_f_ (GPa)		
	C	B	W	C	B	W	C	B	W
PO1	19.42	17.09	16.51	6.90	3.87	2.22	1.43	0.59	1.44
PO2	20.90	19.30	15.10	3.74	3.99	2.49	4.00	1.84	1.67
PO3	22.89	21.73	22.54	3.94	7.17	3.49	1.90	9.62	4.49
PO4	31.57	35.25	34.32	2.66	2.18	3.31	2.47	2.60	11.92
PO5	28.60	42.16	33.51	5.95	2.98	3.56	6.43	11.28	14.59
PO6	33.08	48.21	36.08	7.36	2.77	3.98	10.64	3.79	12.67

**Table 8:** Flexural strength.

0 Days	F_max_ (kN)			σ_max_ (MPa)			ε_max_ (%)			E_f_ (GPa)		
	C	B	W	C	B	W	C	B	W	C	B	W
PO1	0.10	0.11	0.10	46.00	49.88	48.69	7.10	8.40	6.40	1.55	1.71	1.70
PO2	0.12	0.12	0.12	57.98	56.68	56.91	5.50	6.10	6.70	2.03	2.03	2.05
PO3	0.13	0.13	0.12	68.50	64.41	63.99	5.80	6.10	5.30	2.40	2.23	2.29
PO4	0.15	0.15	0.15	68.90	69.17	70.20	4.79	9.25	6.71	2.59	2.63	2.56
PO5	0.15	0.17	0.17	69.78	75.85	77.20	4.39	6.41	5.04	2.58	2.82	2.74
PO6	0.14	0.10	0.15	72.06	46.00	75.08	4.98	7.10	6.13	2.56	1.55	2.58
45 Days	F_max_ (kN)			σ_max_ (MPa)			ε_max_ (%)			E_f_ (GPa)		
	C	B	W	C	B	W	C	B	W	C	B	W
PO1	0.07	0.07	0.07	41.79	36.66	34.96	13.65	15.15	12.78	1.76	1.40	1.32
PO2	0.09	0.09	0.09	49.09	43.89	43.20	10.60	12.61	10.74	2.06	1.58	1.68
PO3	0.09	0.08	0.09	44.56	44.74	48.76	11.57	10.53	16.04	1.77	1.92	1.93
PO4	0.11	0.11	0.11	50.57	51.98	48.26	8.84	10.35	14.54	2.03	2.13	1.74
PO5	0.12	0.12	0.12	56.29	57.25	56.20	8.94	11.53	9.80	2.09	2.28	2.21
PO6	0.11	0.12	0.12	53.82	57.87	53.40	10.39	13.08	12.71	1.95	2.11	1.92
90 Days	F_max_ (kN)			σ_max_ (MPa)			ε_max_ (%)			E_f_ (GPa)		
	C	B	W	C	B	W	C	B	W	C	B	W
PO1	0.10	0.11	0.11	49.46	54.09	54.44	6.09	5.81	7.57	1.73	1.94	1.98
PO2	0.13	0.13	0.13	63.65	64.74	64.25	5.53	4.59	5.38	2.28	2.38	2.16
PO3	0.13	0.14	0.13	74.71	66.69	69.75	4.82	5.42	5.17	2.82	2.22	2.37
PO4	0.16	0.17	0.17	78.25	75.85	79.35	7.42	6.41	6.72	3.00	2.82	2.92
PO5	0.17	0.18	0.18	77.95	90.09	83.86	4.27	4.80	4.92	2.85	3.28	2.94
PO6	0.16	0.17	0.17	77.12	85.52	86.53	4.75	4.49	5.43	2.69	3.07	2.99

**Table 9:** Charpy impact test results.

0 Days	A (mm^2^)			E (J)			U_t_ (kJ/m^2^)		
	C	W	B	C	W	B	C	W	B
PO1	39.92	39.30	40.00	0.20	0.30	0.47	5.01	7.63	11.75
PO2	39.72	40.28	40.24	0.28	0.31	0.50	7.05	7.70	12.43
PO3	37.99	39.04	40.12	0.32	0.32	0.43	8.42	8.20	10.72
PO4	39.98	39.88	37.86	0.29	0.40	0.40	7.25	10.03	10.57
PO5	40.42	40.70	40.32	0.24	0.40	0.49	5.94	9.83	12.15
PO6	38.47	39.14	39.52	0.20	0.41	0.48	5.20	10.48	12.15
45 Days	A (mm^2^)			E (J)			U_t_ (kJ/m^2^)		
	C	W	B	C	W	B	C	W	B
PO1	38.53	39.34	39.56	0.21	0.30	0.42	5.45	7.63	10.62
PO2	39.34	39.88	39.60	0.21	0.47	0.50	5.34	11.79	12.63
PO3	37.90	38.49	38.35	0.30	0.49	0.46	7.92	12.73	11.99
PO4	39.20	39.52	40.08	0.28	0.40	0.47	7.14	10.12	11.73
PO5	39.86	40.64	40.18	0.32	0.50	0.53	8.03	12.30	13.19
PO6	38.56	38.92	39.06	0.34	0.49	0.65	8.82	12.59	16.64
90 Days	A (mm^2^)			E (J)			U_t_ (kJ/m^2^)		
	C	W	B	C	W	B	C	W	B
PO1	36.26	36.63	35.64	0.25	0.43	0.38	6.89	11.74	10.66
PO2	35.64	36.26	36.26	0.27	0.48	0.40	7.58	13.24	11.03
PO3	36.00	34.92	35.89	0.28	0.41	0.39	7.78	11.74	10.87
PO4	37.00	37.24	37.00	0.31	0.46	0.43	8.38	12.35	11.62
PO5	36.63	35.89	37.24	0.32	0.46	0.46	8.74	12.82	12.35
PO6	35.89	36.00	37.37	0.34	0.57	0.52	9.47	15.83	13.91

**Table 10:** Cantilevered drone arm test results.

Specimen	Condition	Load (N)	Deflection (mm)
C	0 days	104.0	48.5
W	0 days	116.0	34.0
B	0 days	108.5	49.6
C	45 days	85.5	32.8
W	45 days	83.5	32.3
B	45 days	90.5	34.6
C	90 days	96.0	31.2
B	90 days	99.0	30.5
W	90 days	99.5	30.3

## Data Availability

The original contributions presented in this study are included in the article. Further inquiries can be directed to the corresponding author.

## Supplementary Materials

The following supporting information can be downloaded at: https://www.mdpi.com/article/10.3390/polym18080963/s1.

## Abbreviations

AM	Additive manufacturing
PLA	Polylactic acid
PVC	Polyvinyl chloride
RM	Reinforcement material
FDM	Fused Deposition Modeling
WM	Wire mesh
FG	Fiber Glass
