# Supplementary material for: Mechanical Performance and Artificial Aging Behavior of Reinforced 3D-Printed PLA Structures for Drone Arm Application
Source: Polymers (Basel). 2026 Apr 15;18(8):963. doi: 10.3390/polym18080963 (PMC13120529; doi:10.3390/polym18080963)
Supplement: Supplementary file 1 [file polymers-18-00963-s001.zip › polymers-4234996-supplementary/S3 -Tables S1-S4.pdf]

Supplementary Tables

Table S1 – Tensile Test (Raw values for Force from the tensile test report)

Tensile stress and break force values are related through the specimen cross-sectional area (≈32 mm²).

Sample	Material	Aging	Break Force (N)	Std Dev (N)	σmax (MPa)	SD σ (MPa)
PO1	C	0 days	490	3.20	15.30	0.10
PO1	B	0 days	630	4.80	19.68	0.15
PO1	W	0 days	628	5.80	19.62	0.18
PO2	C	0 days	639	6.70	19.98	0.21
PO2	B	0 days	699	4.80	21.85	0.15
PO2	W	0 days	664	6.70	20.74	0.21
PO3	C	0 days	699	2.90	21.83	0.09
PO3	B	0 days	676	3.52	21.12	0.11
PO3	W	0 days	699	5.13	21.84	0.16
PO4	C	0 days	1331	5.85	41.59	0.18
PO4	B	0 days	1128	7.70	35.25	0.24
PO4	W	0 days	1086	7.05	33.94	0.22
PO5	C	0 days	1259	9.95	39.34	0.31
PO5	B	0 days	1111	3.22	34.71	0.10
PO5	W	0 days	1267	4.24	39.58	0.13
PO6	C	0 days	1416	4.83	44.24	0.15
PO6	B	0 days	1216	7.74	38.01	0.24
PO6	W	0 days	1573	7.05	49.16	0.22
PO1	C	45 days	604	9.91	18.88	0.31

PO1	B	45 days	510	7.01	15.95	0.22
PO1	W	45 days	609	3.25	19.02	0.10
PO2	C	45 days	579	5.1	18.09	0.16
PO2	B	45 days	576	5.8	18.01	0.18
PO2	W	45 days	768	7.7	23.99	0.24
PO3	C	45 days	645	3.5	20.16	0.11
PO3	B	45 days	675	11.2	21.10	0.35
PO3	W	45 days	652	6.7	20.38	0.21
PO4	C	45 days	933	8.0	29.17	0.25
PO4	B	45 days	1151	4.8	35.97	0.15
PO4	W	45 days	1082	5.8	33.82	0.18
PO5	C	45 days	922	4.5	28.82	0.14
PO5	B	45 days	1217	8.3	38.04	0.26
PO5	W	45 days	781	8.6	24.42	0.27
PO6	C	45 days	939	9.3	29.35	0.29
PO6	B	45 days	1438	7.0	44.94	0.22
PO6	W	45 days	1105	6.7	34.53	0.21
PO1	C	90 days	621	3.5	19.42	0.11
PO1	B	90 days	547	5.8	17.09	0.18
PO1	W	90 days	528	4.5	16.51	0.14
PO2	C	90 days	669	6.1	20.90	0.19
PO2	B	90 days	618	3.2	19.30	0.10
PO2	W	90 days	483	4.8	15.10	0.15
PO3	C	90 days	732	2.2	22.89	0.07
PO3	B	90 days	695	6.7	21.73	0.21
PO3	W	90 days	721	7.0	22.54	0.22

PO4	C	90 days	1010	3.5	31.57	0.11
PO4	B	90 days	1128	5.1	35.25	0.16
PO4	W	90 days	1098	5.8	34.32	0.18
PO5	C	90 days	915	2.9	28.60	0.09
PO5	B	90 days	1349	5.4	42.16	0.17
PO5	W	90 days	1072	7.7	33.51	0.24
PO6	C	90 days	1059	6.4	33.08	0.20
PO6	B	90 days	1543	9.9	48.21	0.31
PO6	W	90 days	1155	7.4	36.08	0.23

Table S2 – Bending Test

Maximum force values were obtained from three-point bending tests. Flexural stress standard deviations were calculated from force deviations using the bending stress relationship.

Sample	Material	Aging	Break Force (N)	Std Dev (N)	σmax (MPa)	SD σ (MPa)
PO1	C	0 days	96	6.1	46.00	2.92
PO1	B	0 days	106	5.5	49.88	2.59
PO1	W	0 days	102	3.2	48.69	1.53
PO2	C	0 days	119	6.2	57.98	3.02
PO2	B	0 days	121	7.7	56.68	3.61
PO2	W	0 days	119	9.1	56.91	4.36
PO3	C	0 days	127	4.2	68.50	2.27
PO3	B	0 days	127	3.8	64.41	1.93
PO3	W	0 days	124	3.5	63.99	1.81
PO4	C	0 days	147	9.9	68.90	4.64
PO4	B	0 days	146	7.5	69.17	3.55

PO4	W	0 days	150	6.5	70.2	3.04
PO5	C	0 days	152	9.2	69.78	4.22
PO5	B	0 days	169	6.5	75.85	2.91
PO5	W	0 days	169	11.4	77.20	5.21
PO6	C	0 days	143	11.2	72.06	5.64
PO6	B	0 days	96	5.3	46.00	2.54
PO6	W	0 days	151	4.9	75.08	2.44
PO1	C	45 days	74	7.8	41.79	4.46
PO1	B	45 days	74	9.5	36.66	4.71
PO1	W	45 days	72	11.0	34.96	5.34
PO2	C	45 days	91	8.7	49.09	4.69
PO2	B	45 days	94	6.6	43.89	3.08
PO2	W	45 days	88	7.9	43.2	3.88
PO3	C	45 days	88	9.8	44.56	4.96
PO3	B	45 days	83	5.2	44.74	2.86
PO3	W	45 days	90	5.5	48.76	2.98
PO4	C	45 days	107	6.9	50.57	3.26
PO4	B	45 days	111	8.8	51.98	4.12
PO4	W	45 days	105	9.8	48.26	4.51
PO5	C	45 days	121	4.5	56.29	2.09
PO5	B	45 days	124	4.7	57.25	2.17
PO5	W	45 days	124	8.8	56.20	3.99
PO6	C	45 days	111	9.8	53.82	4.75
PO6	B	45 days	119	3.5	57.87	1.72
PO6	W	45 days	117	4.9	53.40	2.24
PO1	C	90 days	98	5.1	49.46	2.57

PO1	B	90 days	110	7.2	54.09	3.54
PO1	W	90 days	109	7.9	54.44	3.95
PO2	C	90 days	125	7.9	63.65	4.02
PO2	B	90 days	131	7.4	64.74	3.66
PO2	W	90 days	130	3.9	64.25	1.93
PO3	C	90 days	129	5.9	74.71	3.41
PO3	B	90 days	137	9.8	66.69	4.77
PO3	W	90 days	134	9.9	69.75	5.16
PO4	C	90 days	157	10.5	78.25	5.23
PO4	B	90 days	169	6.9	75.85	3.10
PO4	W	90 days	166	4.3	79.35	2.05
PO5	C	90 days	166	8.6	77.95	4.04
PO5	B	90 days	183	12.1	90.09	5.95
PO5	W	90 days	179	13.1	83.86	6.14
PO6	C	90 days	156	5.2	77.12	2.57
PO6	B	90 days	166	5.1	85.52	2.63
PO6	W	90 days	167	8.5	86.53	4.41

Table S3 – Charpy Impact Test (Energy in J)

The standard deviation of impact strength (Ut) was calculated from the standard deviation of absorbed energy (E) using error propagation.

Sample	Material	Aging	E (J)	Std Dev (J)	Ut (kJ/m^2)	Std Dev (kJ/m2)
PO1	C	0 days	0.20	0.036	5.01	0.90
PO1	B	0 days	0.30	0.027	7.63	0.69
PO1	W	0 days	0.47	0.029	11.75	0.72
PO2	C	0 days	0.28	0.045	7.05	1.13

PO2	B	0 days	0.31	0.037	7.70	0.92
PO2	W	0 days	0.50	0.022	12.43	0.55
PO3	C	0 days	0.32	0.029	8.42	0.76
PO3	B	0 days	0.32	0.035	8.20	0.90
PO3	W	0 days	0.43	0.041	10.72	1.02
PO4	C	0 days	0.29	0.019	7.25	0.47
PO4	B	0 days	0.40	0.031	10.03	0.78
PO4	W	0 days	0.40	0.019	10.57	0.50
PO5	C	0 days	0.24	0.025	5.94	0.62
PO5	B	0 days	0.4	0.027	9.83	0.66
PO5	W	0 days	0.49	0.029	12.15	0.72
PO6	C	0 days	0.2	0.034	5.20	0.88
PO6	B	0 days	0.41	0.037	10.48	0.95
PO6	W	0 days	0.48	0.032	12.15	0.81
PO1	C	45 days	0.21	0.021	5.45	0.55
PO1	B	45 days	0.30	0.034	7.63	0.87
PO1	W	45 days	0.42	0.022	10.62	0.56
PO2	C	45 days	0.21	0.015	5.34	0.38
PO2	B	45 days	0.47	0.019	11.79	0.48
PO2	W	45 days	0.50	0.027	12.63	0.68
PO3	C	45 days	0.30	0.045	7.92	1.19
PO3	B	45 days	0.49	0.032	12.73	0.83
PO3	W	45 days	0.46	0.031	11.99	0.81
PO4	C	45 days	0.28	0.018	7.14	0.46
PO4	B	45 days	0.40	0.019	10.12	0.48
PO4	W	45 days	0.47	0.016	11.73	0.40

PO5	C	45 days	0.32	0.019	8.03	0.48
PO5	B	45 days	0.5	0.015	12.3	0.37
PO5	W	45 days	0.53	0.027	13.19	0.67
PO6	C	45 days	0.34	0.031	8.82	0.80
PO6	B	45 days	0.49	0.029	12.59	0.75
PO6	W	45 days	0.65	0.045	16.64	1.15
PO1	C	90 days	0.25	0.012	6.89	0.33
PO1	B	90 days	0.43	0.017	11.74	0.46
PO1	W	90 days	0.38	0.030	10.66	0.84
PO2	C	90 days	0.27	0.022	7.58	0.62
PO2	B	90 days	0.48	0.033	13.24	0.91
PO2	W	90 days	0.4	0.031	11.03	0.85
PO3	C	90 days	0.28	0.036	7.78	1.00
PO3	B	90 days	0.41	0.038	11.74	1.09
PO3	W	90 days	0.39	0.034	10.87	0.95
PO4	C	90 days	0.31	0.021	8.38	0.57
PO4	B	90 days	0.46	0.018	12.35	0.48
PO4	W	90 days	0.43	0.040	11.62	1.08
PO5	C	90 days	0.32	0.026	8.74	0.71
PO5	B	90 days	0.46	0.022	12.82	0.61
PO5	W	90 days	0.46	0.029	12.35	0.78
PO6	C	90 days	0.34	0.027	9.47	0.75
PO6	B	90 days	0.57	0.026	15.83	0.72
PO6	W	90 days	0.52	0.031	13.91	0.83

Table S4 – Cantilever Test (Max Load in N)

Sample	Material	Aging	Value	Std Dev
PO3	C	0 days	104.0	3.49
PO3	B	0 days	116.0	4.50
PO3	W	0 days	108.5	4.12
PO3	C	45 days	85.5	2.52
PO3	B	45 days	83.5	2.49
PO3	W	45 days	90.5	3.30
PO3	C	90 days	96.0	3.15
PO3	B	90 days	99.0	4.10
PO3	W	90 days	99.5	5.10

Note: Values represent mean ± standard deviation of directly measured quantities (force and absorbed energy). Derived parameters are included and calculated from measured quantities.
