# Supplementary material for: Mechanical Performance and Artificial Aging Behavior of Reinforced 3D-Printed PLA Structures for Drone Arm Application
Source: Polymers (Basel). 2026 Apr 15;18(8):963. doi: 10.3390/polym18080963 (PMC13120529; doi:10.3390/polym18080963)
Supplement: Supplementary file 1 [file polymers-18-00963-s001.zip › polymers-4234996-supplementary/S4-Tables S5-S33.pdf]

MICROSCOPY

Table S5. Microscopic images of surface before and after artificial ageing – C specimens

Sample	Before agenig	45 days	90 days
PO1 C	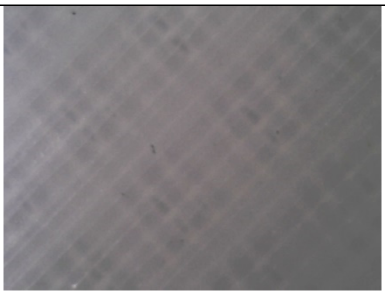	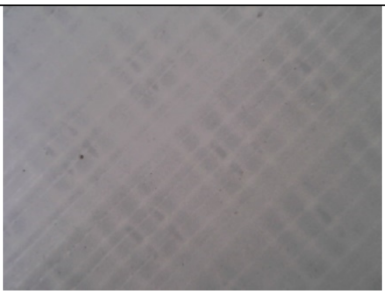	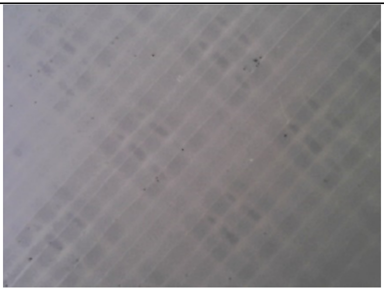
PO2 C	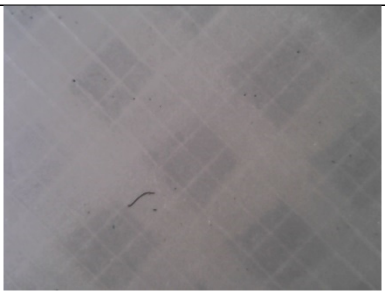	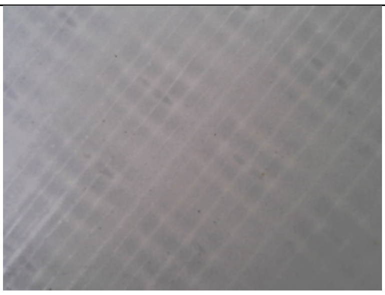	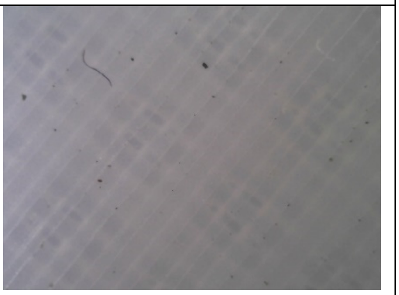
PO3 C	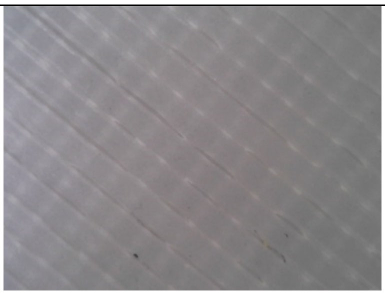	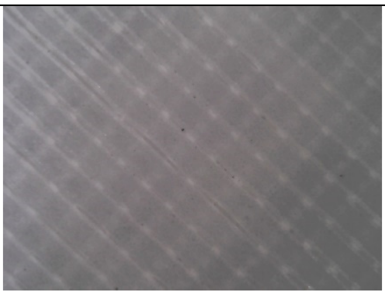	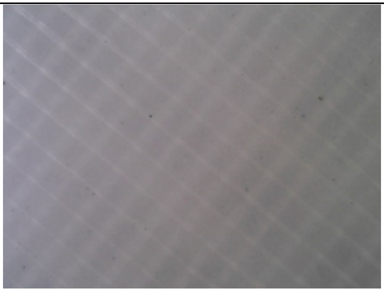
PO4 C	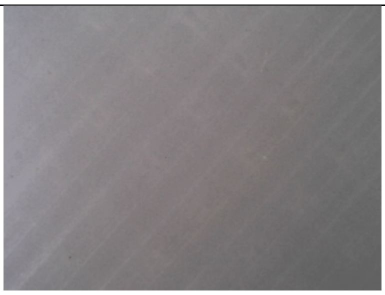	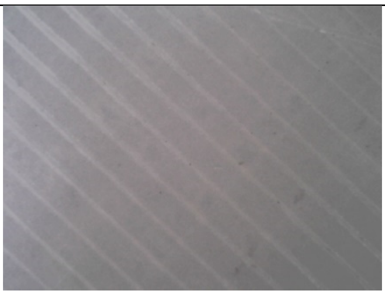	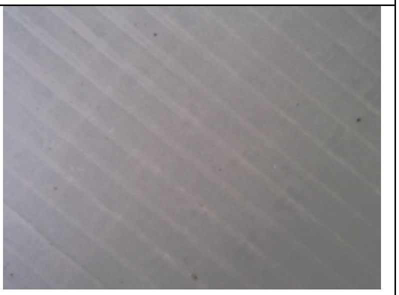
PO5 C	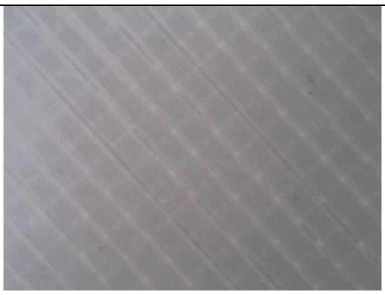	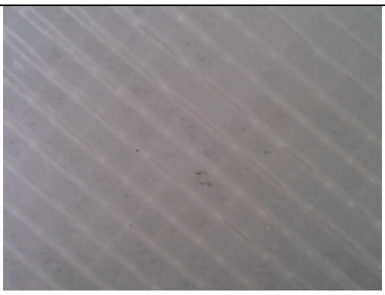	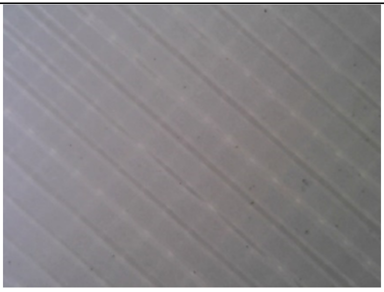
PO6 C	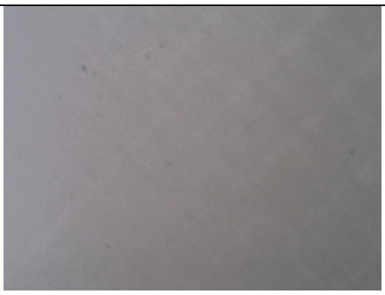	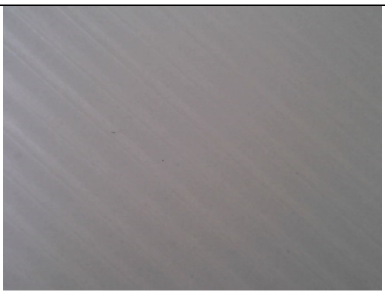	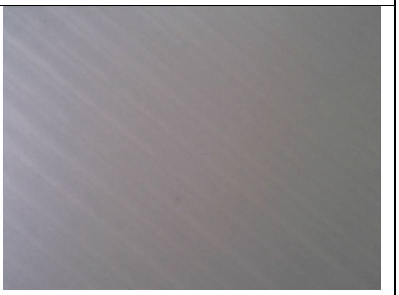

COLORIMETRY

Table S6. Results of colorimetric analysis before ageing – C specimens

Sample	L*	a*	b*	Pseudo-color
PO1 C	80,98	-0,55	-1,27	
PO2 C	82,83	-0,46	-1,35	
PO3 C	82,66	-0,64	-1,24	
PO4 C	84,58	-1,21	-1,43	
PO5 C	84,57	-1,18	-0,88	
PO6 C	84,43	-1,19	-1,74	

Table S7. Results of colorimetric analysis after 45 days of artificial ageing – C specimens

Sample	L*	a*	b*	Pseudo-color
PO1 C	81,21	-0,01	-1,74	
PO2 C	81,38	0,11	-1,94	
PO3 C	82,67	-0,02	-1,79	
PO4 C	84,41	-0,52	-2,07	
PO5 C	83,14	-0,59	-1,75	
PO6 C	84,81	-0,39	-2,28	

Table S8. Results of colorimetric analysis after 90 days of artificial ageing – C specimens

Sample	L*	a*	b*	Pseudo-color
PO1 C	83,16	-0,69	-2,09	
PO2 C	82,96	-0,59	-1,84	
PO3 C	83,22	-0,65	-2,46	
PO4 C	85,37	-0,70	-2,23	
PO5 C	85,52	-0,93	-1,72	
PO6 C	85,52	-0,62	-2,59	

Table S9. delta E – C specimens

Sample	delta E 45	delta E 90
PO1 C	0,75	2,33
PO2 C	1,67	0,52
PO3 C	0,83	1,34
PO4 C	0,96	1,23
PO5 C	1,77	1,29
PO6 C	1,04	1,49

CONTACT ANGLE AND SURFACE FREE ENERGY

Table S10. Results of contact angle measurement before ageing – C specimens

Sample	Image acquision possition	Contact angle value	Surface free energy (mJ/m^2)
PO1 C	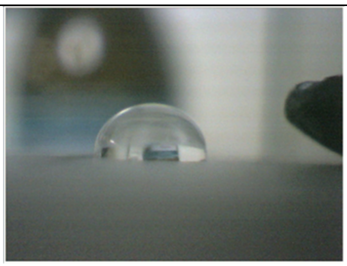	89.85	23.54
PO2 C	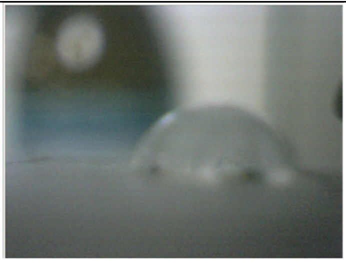	90.95	22.93
PO3 C	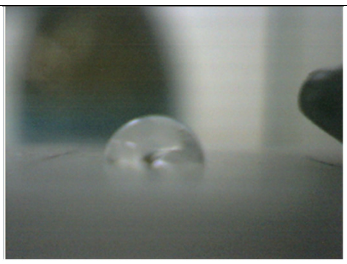	91.79	22.47
PO4 C	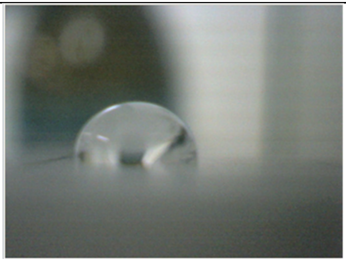	92.00	22.36
PO5 C	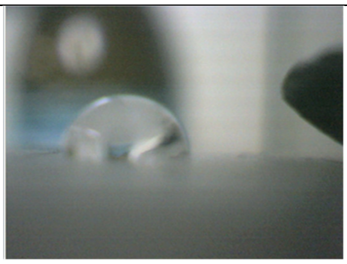	91.83	22.45
PO6 C	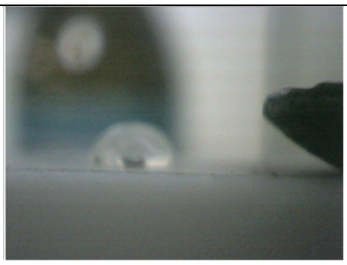	85.73	25.83

Table S11. Results of contact angle measurement after 45 days of artificial ageing – C specimens

Sample	Image acquision possition	Contact angle value	Surface free energy (mJ/m^2)
PO1 C	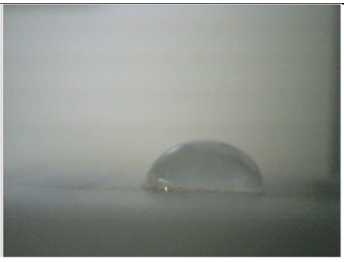	83.02	27.36
PO2 C	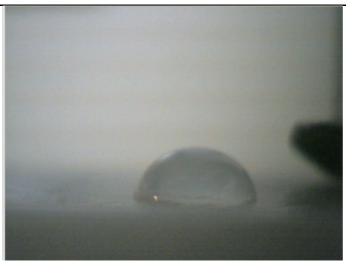	84.28	26.65
PO3 C	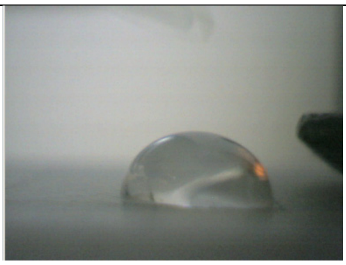	89.60	23.68
PO4 C	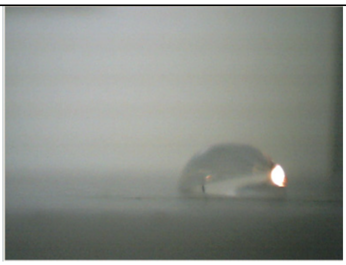	94.36	21.08
PO5 C	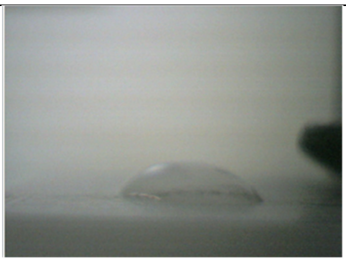	58.90	41.17
PO6 C	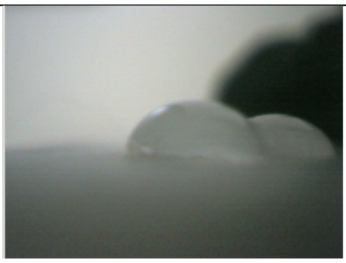	79.09	29.59

Table S12. Results of contact angle measurement after 90 days of artificial ageing – C specimens

Sample	Image acquision possition	Contact angle value	Surface free energy (mJ/m^2)
PO1 C	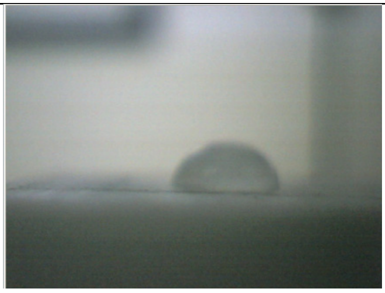	87.25	24.98
PO2 C	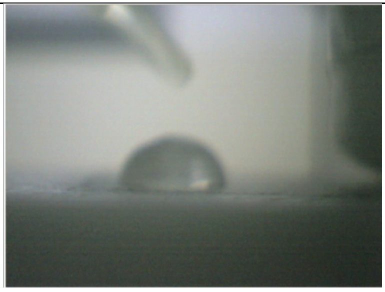	93.16	21.73
PO3 C	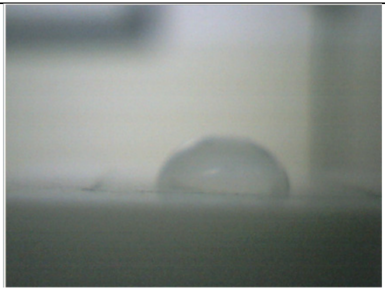	82.87	27.44
PO4 C	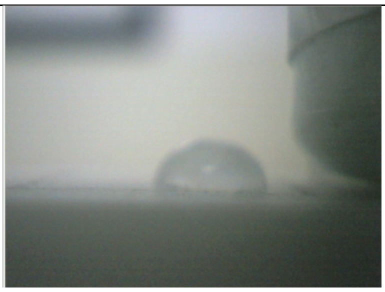	86.65	25.32
PO5 C	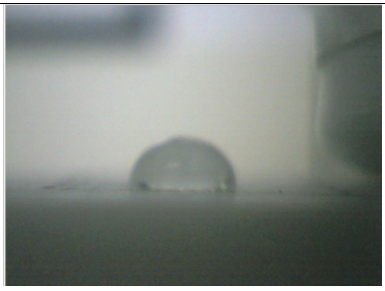	89.99	23.46
PO6 C	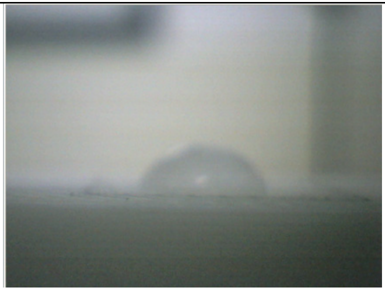	73.39	32.83

Table S13. Comparative results of contact angle measurements – C specimens

Sample	Before ageing	After 45 days	After 90 days
PO1 C	89.85	83.02	87.25
PO2 C	90.95	84.28	93.16
PO3 C	91.79	89.60	82.87
PO4 C	92.00	94.36	86.65
PO5 C	91.83	58.90	89.99
PO6 C	85.73	79.09	73.39

MICROSCOPY

Table S14. Microscopic images of samples surface before and after artificial ageing – B specimens

Sample	Before agenig	45 days	90 days
PO1 B	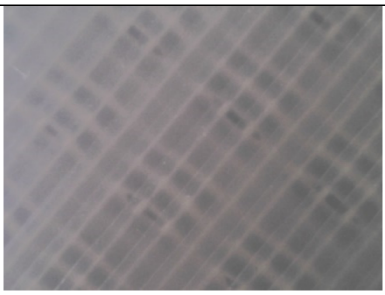	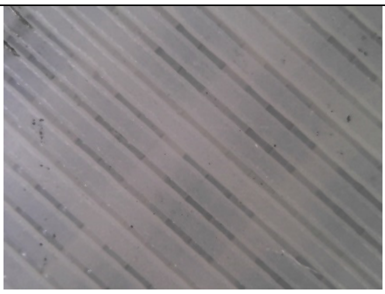	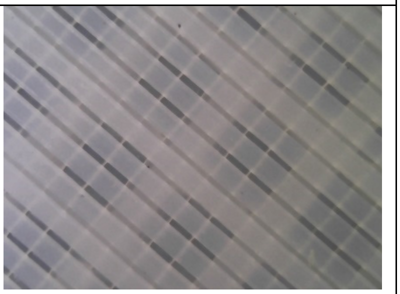
PO2 B	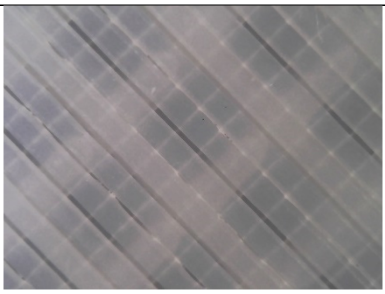	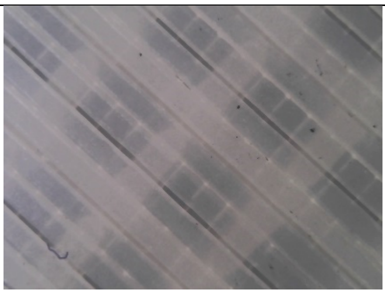	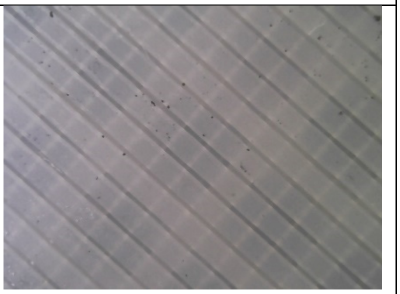
PO3 B	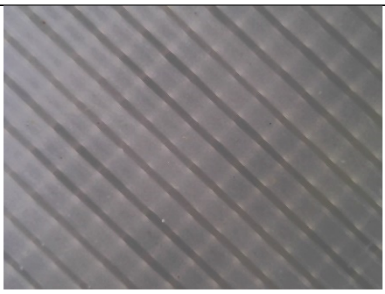	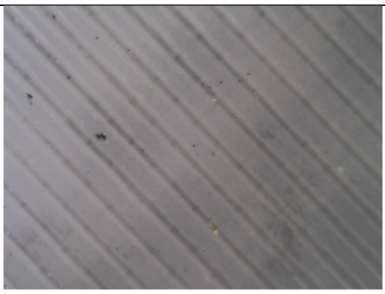	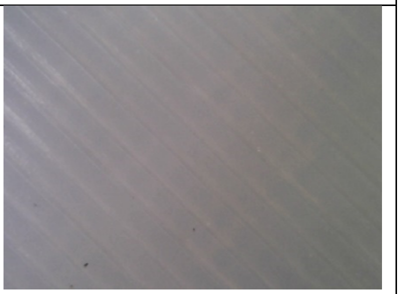
PO4 B	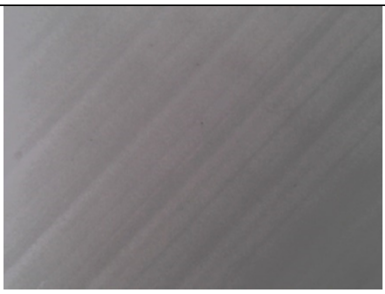	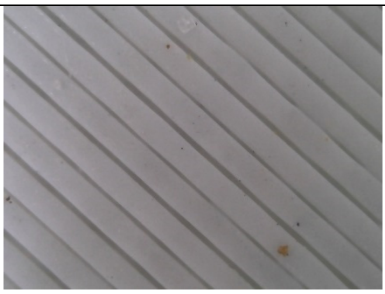	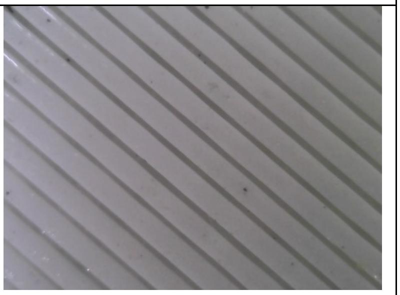
PO5 B	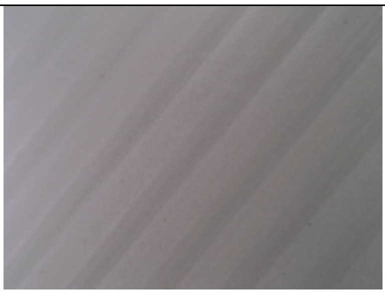	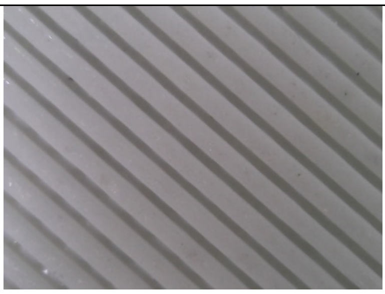	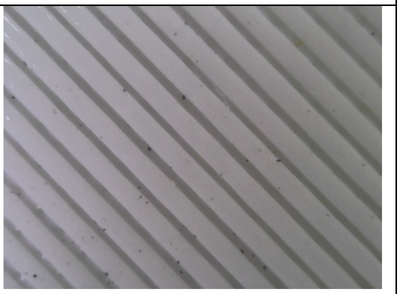
PO6 B	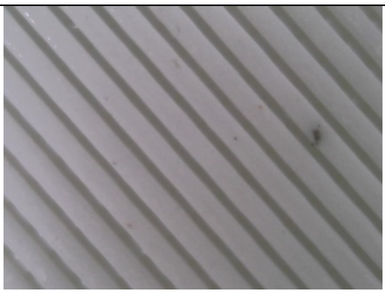	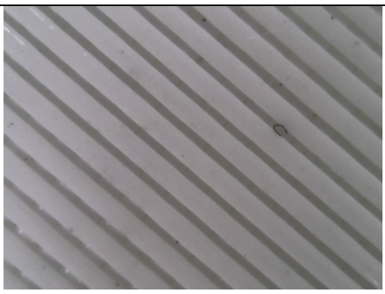	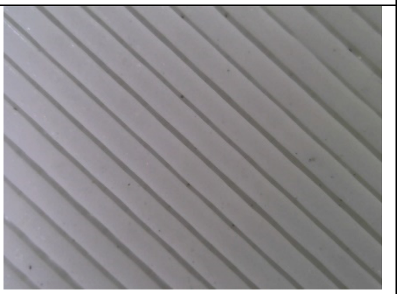

COLORIMETRY

Table S15. Results of colorimetric analysis before ageing – B specimens

Sample	L*	a*	b*	Pseudo-color
PO1 B	77,19	-1,16	-3,72	
PO2 B	77,60	-1,21	-3,63	
PO3 B	79,19	-1,16	-2,87	
PO4 B	94,60	-0,93	2,94	
PO5 B	95,12	-0,94	3,00	
PO6 B	95,65	-0,95	3,40	

Table S16. Results of colorimetric analysis after 45 days of artificial ageing

Sample	L*	a*	b*	Pseudo-color
PO1 B	76,22	-1,11	-3,71	
PO2 B	76,38	-1,14	-3,17	
PO3 B	78,36	-1,29	-2,27	
PO4 B	94,41	-0,85	2,25	
PO5 B	95,33	-0,87	2,44	
PO6 B	95,16	-0,79	2,31	

Table S17. Results of colorimetric analysis after 90 days of artificial ageing

Sample	L*	a*	b*	Pseudo-color
PO1 B	76,99	-1,32	-3,84	
PO2 B	77,81	-1,17	-3,41	
PO3 B	79,71	-0,92	-3,85	
PO4 B	95,19	-0,77	2,09	
PO5 B	95,19	-0,80	1,33	
PO6 B	96,21	-0,79	1,99	

Table S18. delta E – B specimens

Sample	delta E 45	delta E 90
PO1 B	0,97	0,28
PO2 B	1,31	0,31
PO3 B	1,03	1,14
PO4 B	0,72	1,05
PO5 B	0,60	1,68
PO6 B	1,21	1,53

CONTACT ANGLE AND SURFACE FREE ENERGY

Table S19. Results of contact angle measurement before ageing – B specimens

Sample	Image acquision possition	Contact angle value	Surface free energy (mJ/m^2)
PO1 B	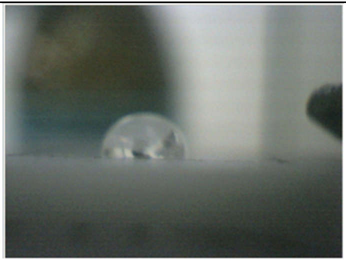	94.36	21.08
PO2 B	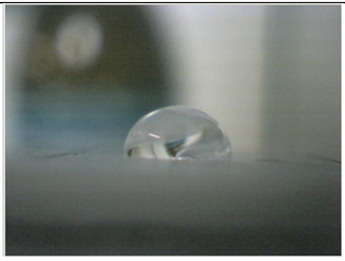	89.12	23.94
PO3 B	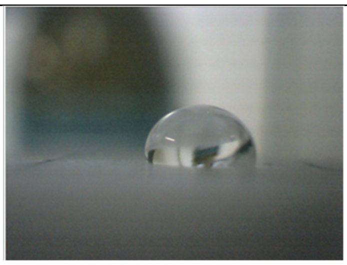	95.98	20.21
PO4 B	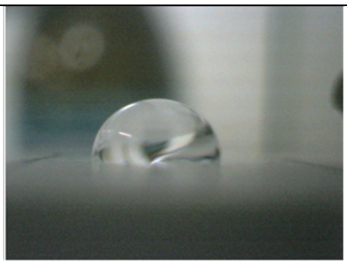	91.60	22.58
PO5 B	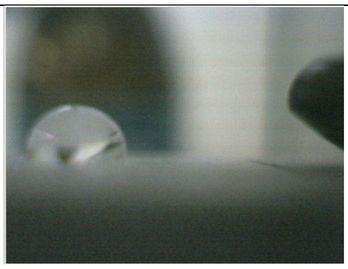	94.73	20.88
PO6 B	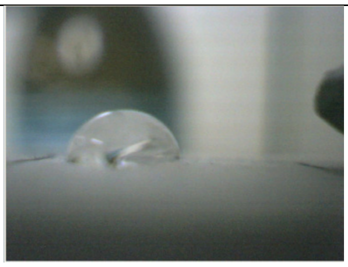	85.73	25.83

Table S20. Results of contact angle measurement after 45 days of artificial ageing – B specimens

Sample	Image acquision possition	Contact angle value	Surface free energy (mJ/m^2)
PO1 B	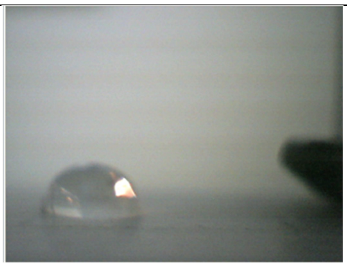	109.30	13.45
PO2 B	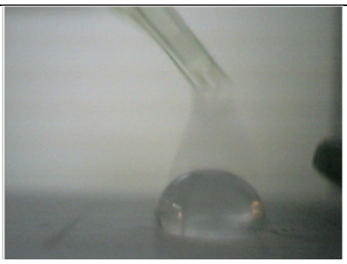	102.06	17.02
PO3 B	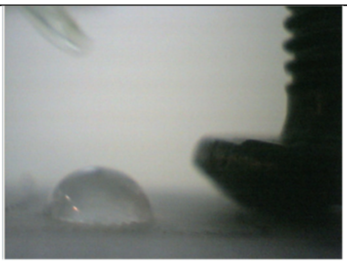	91.95	22.39
PO4 B	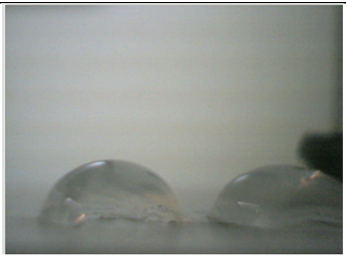	83.01	27.36
PO5 B	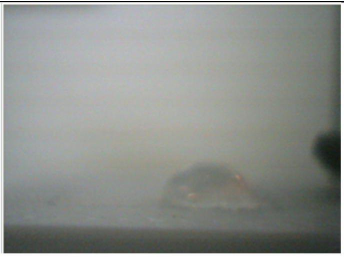	84.57	26.48
PO6 B	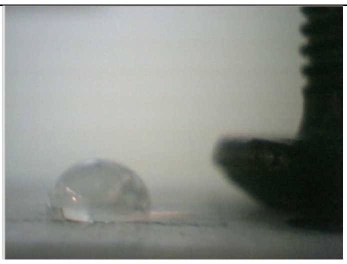	104.10	15.99

Table S21. Results of contact angle measurement after 90 days of artificial ageing– B specimens

Sample	Image acquision possition	Contact angle value	Surface free energy (mJ/m^2)
PO1 B	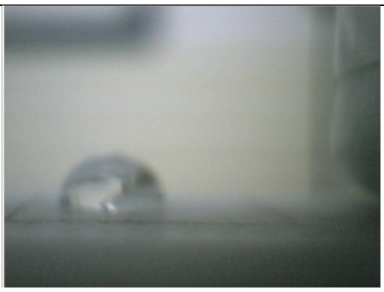	103.67	16.20
PO2 B	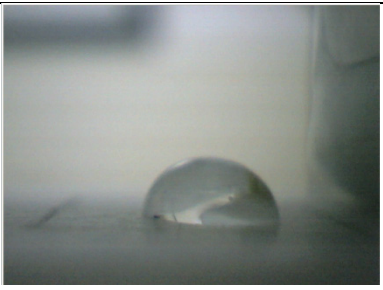	89.80	23.57
PO3 B	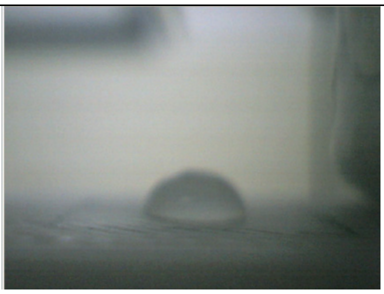	90.57	23.14
PO4 B	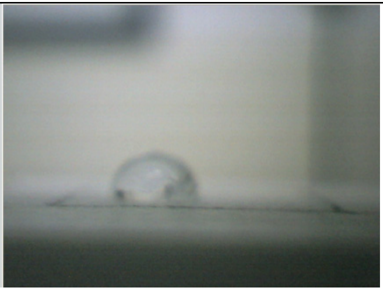	100.80	17.68
PO5 B	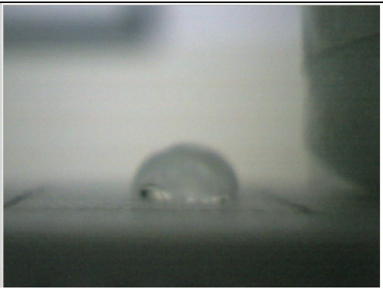	98.89	18.67
PO6 B	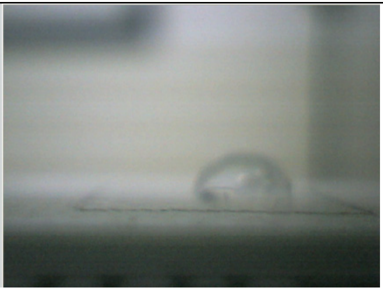	88.03	24.54

Table S22. Comparative results of contact angle measurements– B specimens

Sample	Before ageing	After 45 days	After 90 days
PO1 B	94.36	109.30	103.67
PO2 B	89.12	102.06	89.80
PO3 B	95.98	91.95	90.57
PO4 B	91.60	83.01	100.80
PO5 B	94.73	84.57	98.89
PO6 B	85.73	104.10	88.03

MICROSCOPY

Table S23. Microscopic images of samples surface before and after artificial ageing – W specimens

Sample	Before agenig	45 days	90 days
PO1 W	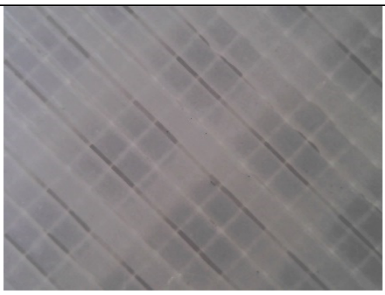	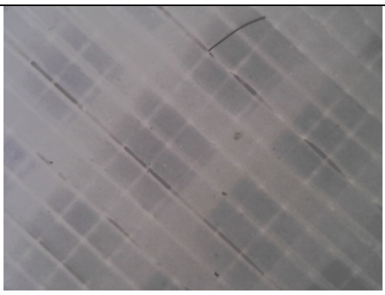	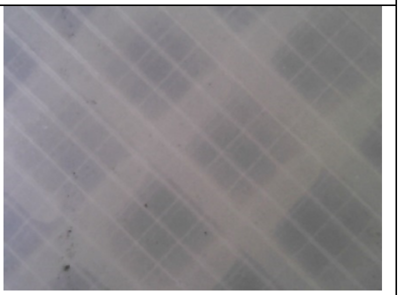
PO2 W	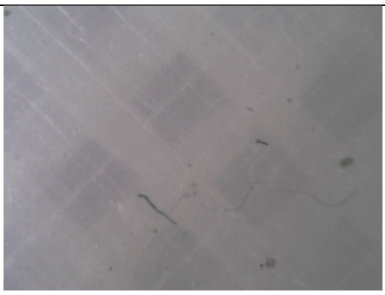	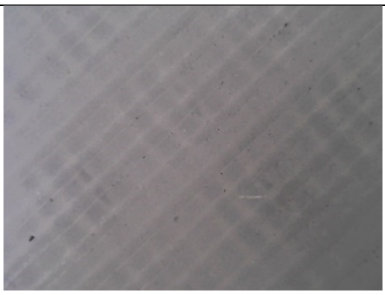	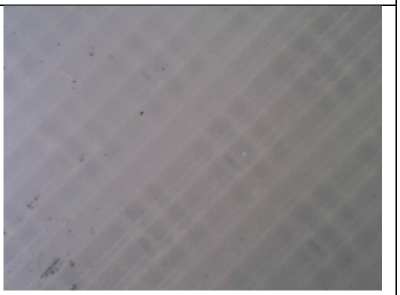
PO3 W	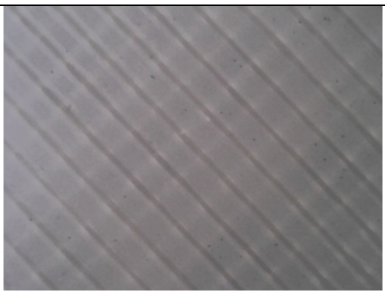	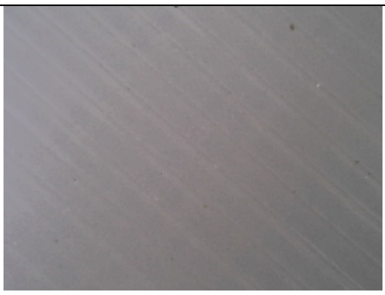	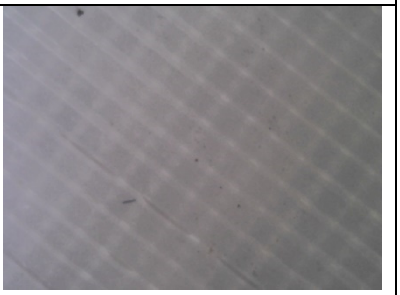
PO4 W	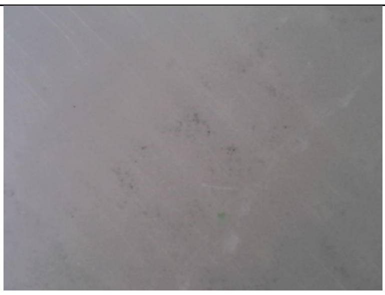	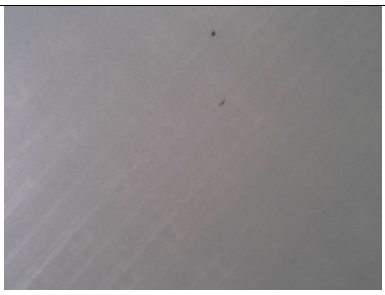	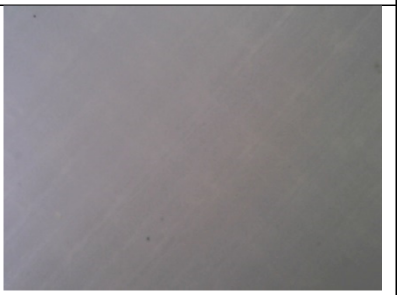
PO5 W	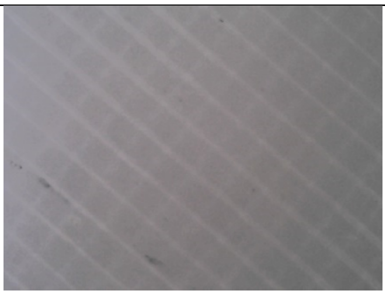	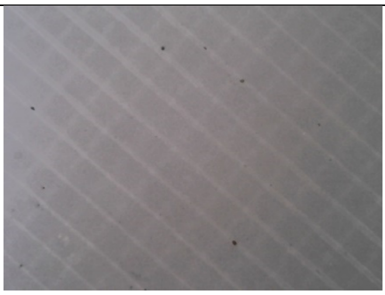	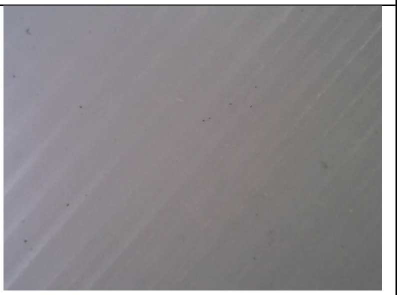
PO6 W	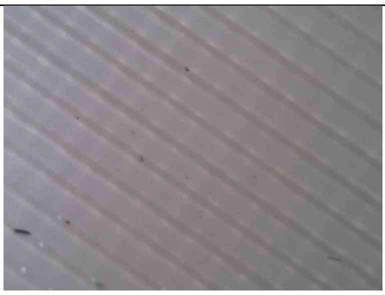	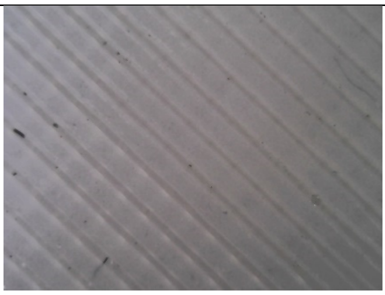	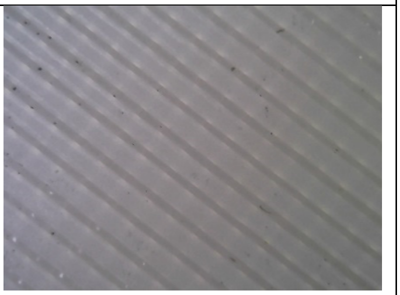

COLORIMETRY

Table S24. Results of colorimetric analysis before ageing– W specimens

Sample	L*	a*	b*	Pseudo-color
PO1 W	81,39	-0,65	-1,50	
PO2 W	83,15	-0,44	-1,17	
PO3 W	84,44	-0,59	-1,03	
PO4 W	84,78	-1,21	-0,86	
PO5 W	84,97	-1,14	-1,14	
PO6 W	84,81	-1,03	-0,98	

Table 25. Results of colorimetric analysis after 45 days of artificial ageing– W specimens

Sample	L*	a*	b*	Pseudo-color
PO1 W	81,29	-0,15	-1,90	
PO2 W	82,24	-0,05	-1,43	
PO3 W	84,51	-0,03	-1,70	
PO4 W	84,18	-0,51	-1,86	
PO5 W	84,48	-0,53	-2,00	
PO6 W	83,03	-0,46	-1,45	

Table 26. Results of colorimetric analysis after 90 days of artificial ageing– W specimens

Sample	L*	a*	b*	Pseudo-color
PO1 W	80,30	-1,17	-3,22	
PO2 W	83,84	-0,48	-1,62	
PO3 W	84,08	-0,53	-1,37	
PO4 W	85,58	-0,86	-2,22	
PO5 W	85,22	-0,90	-1,74	
PO6 W	84,99	-0,92	-1,68	

Table 27. delta E– W specimens

Sample	delta E 45	delta E 90
PO1 W	0,65	2,10
PO2 W	1,02	0,82
PO3 W	0,88	0,50
PO4 W	1,36	1,62
PO5 W	1,16	0,69
PO6 W	1,93	0,73

CONTACT ANGLE AND SURFACE FREE ENERGY

Table 28. Results of contact angle measurement before ageing– W specimens

Sample	Image acquision possition	Contact angle value	Surface free energy (mJ/m^2)
PO1 W	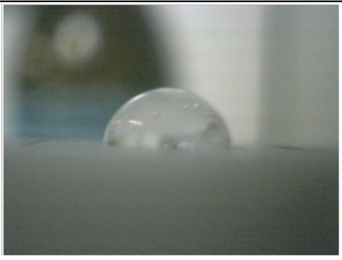	86.18	25.58
PO2 W	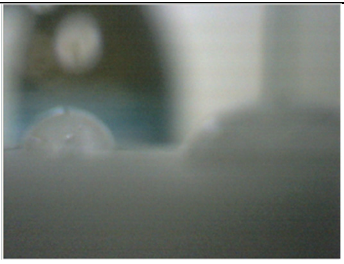	81.22	28.38
PO3 W	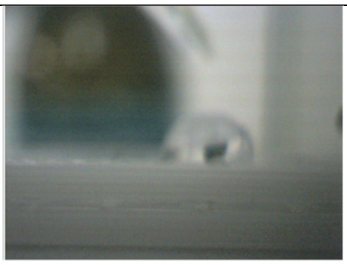	98.28	18.99
PO4 W	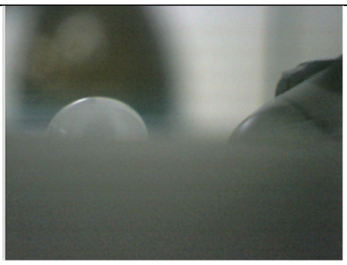	78.82	29.74
PO5 W	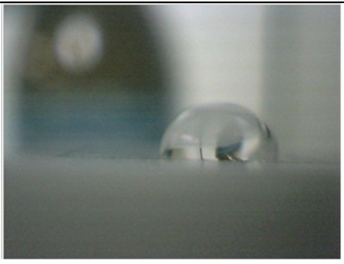	89.22	23.89
PO6 W	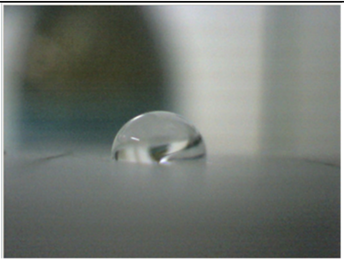	94.11	21.21

Table 29. Results of contact angle measurement after 45 days of artificial ageing– W specimens

Sample	Image acquision possition	Contact angle value	Surface free energy (mJ/m^2)
PO1 W	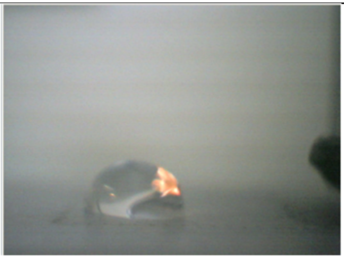	98.81	18.71
PO2 W	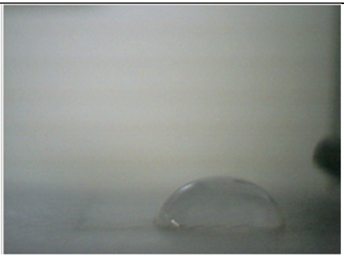	80.68	28.68
PO3 W	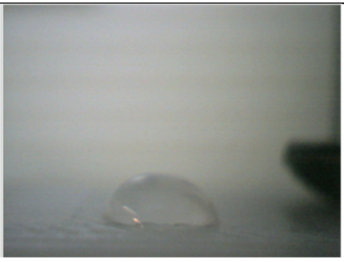	84.67	26.43
PO4 W	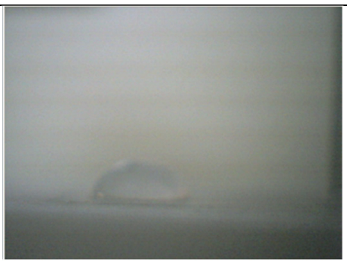	86.19	25.57
PO5 W	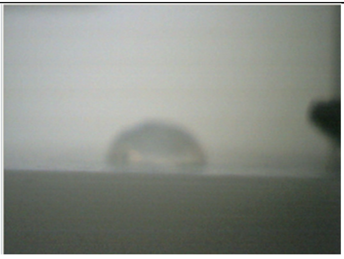	95.35	20.55
PO6 W	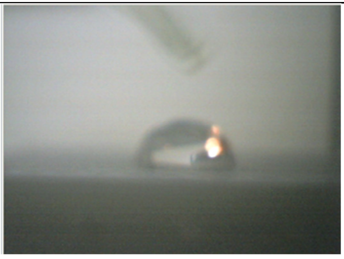	94.09	21.22

Table 30. Results of contact angle measurement after 90 days of artificial ageing– W specimens

Sample	Image acquision possition	Contact angle value	Surface free energy (mJ/m^2)
PO1 W	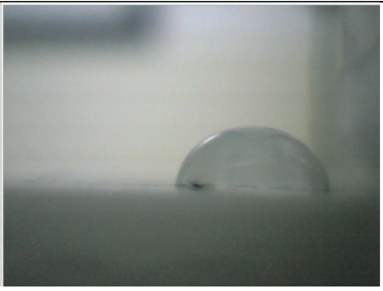	78.06	30.18
PO2 W	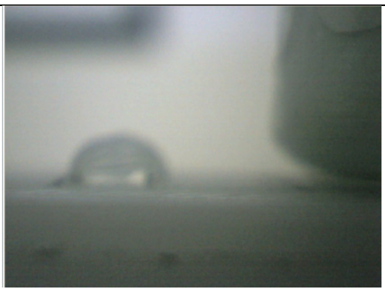	100.96	17.60
PO3 W	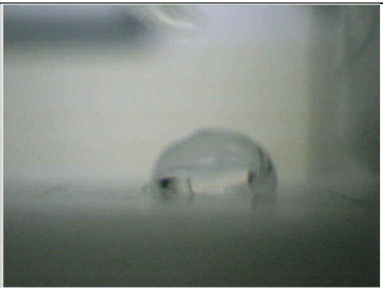	95.93	20.24
PO4 W	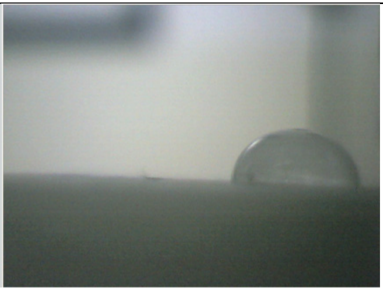	80.91	28.55
PO5 W	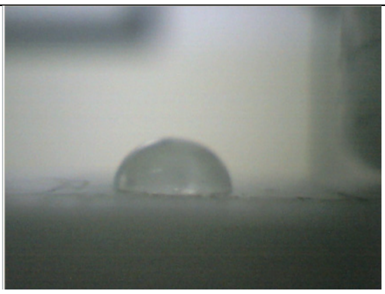	85.30	26.07
PO6 W	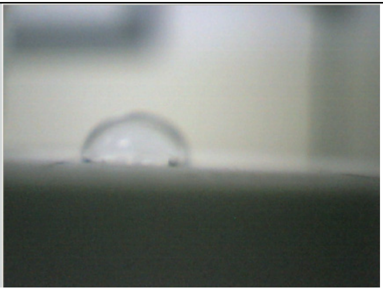	84.65	26.43

Table 31. Comparative results of contact angle measurements– W specimens

Sample	Before ageing	After 45 days	After 90 days
PO1 W	86.18	98.81	78.06
PO2 W	81.22	80.68	100.96
PO3 W	98.28	84.67	95.93
PO4 W	78.82	86.19	80.91
PO5 W	89.22	95.35	85.30
PO6 W	94.11	94.09	84.65

MICROSCOPY

Table 32. Microscopic images of drone arm samples before and after artificial ageing

Drone arm	Before agenig	45 days	90 days
C upper surface	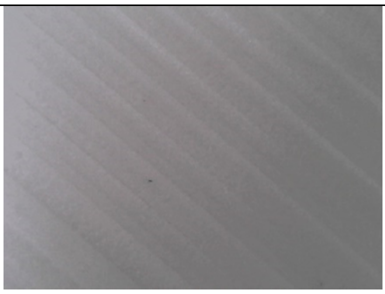	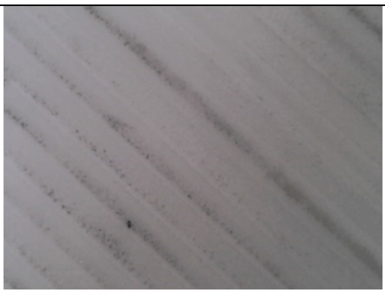	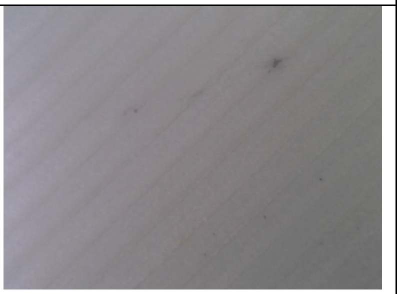
B upper surface	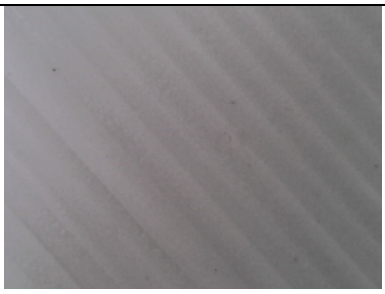	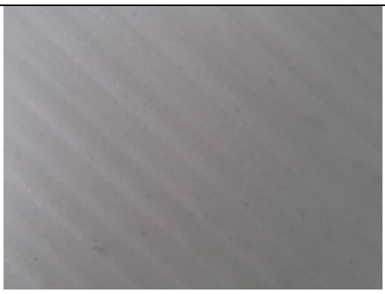	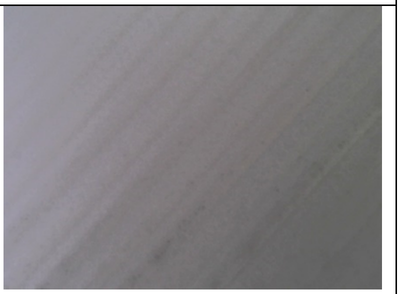
B Side surface	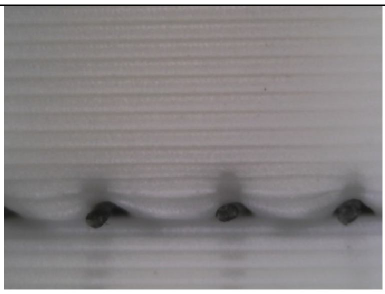	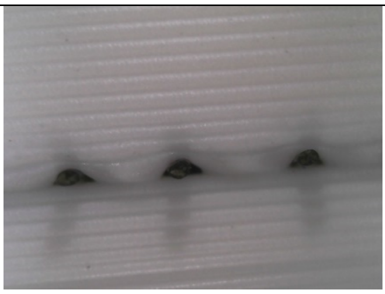	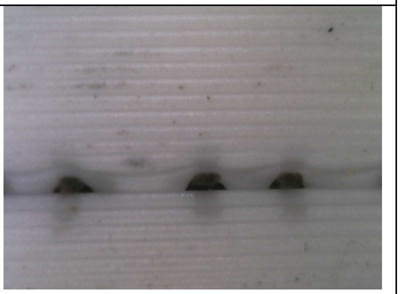
W upper surface	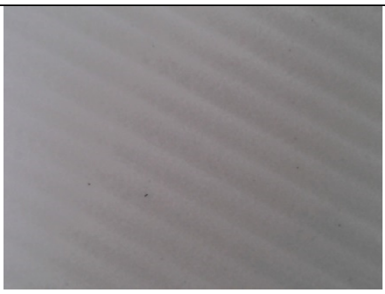	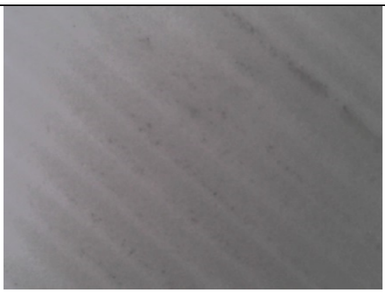	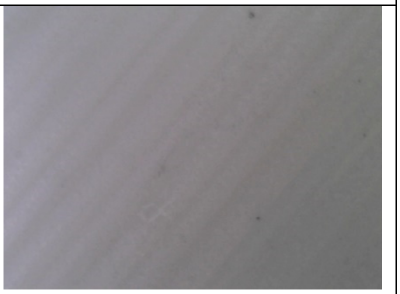
W Side surface	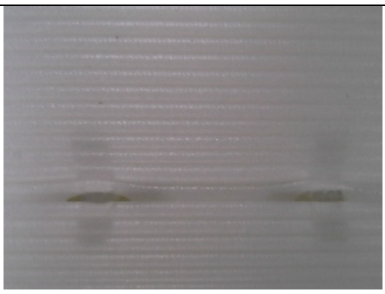	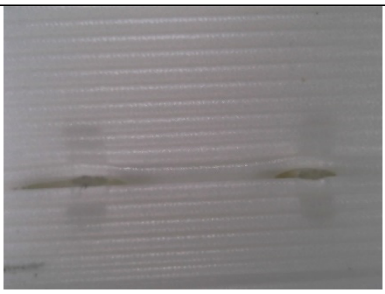	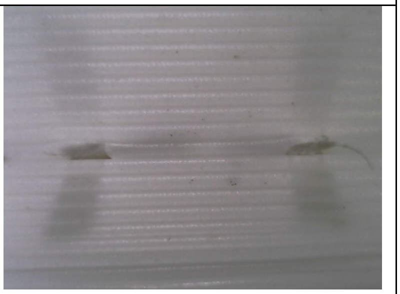

Table 33. Results of colorimetric analysis of drone arm samples before and after artificial ageing

Sample	L*	a*	b*	Delta E	Pseudo-color
C 0	95,83	-0,80	2,36	/	
C 45	94,54	-0,68	1,85	1,39	
C 90	94,48	-0,67	1,60	1,55	
B 0	95,06	-0,84	2,39	/	
B 45	95,33	-0,75	1,70	0,75	
B 90	96,21	-0,68	1,43	1,51	
W 0	95,32	-0,77	2,37	/	
W 45	94,93	-0,72	1,80	0,69	

W 90	96,07	-0,62	1,50	1,16	
