# Supplementary figures and images for: Mechanical Performance and Artificial Aging Behavior of Reinforced 3D-Printed PLA Structures for Drone Arm Application
Source: Polymers (Basel). 2026 Apr 15;18(8):963. doi: 10.3390/polym18080963 (PMC13120529; doi:10.3390/polym18080963)

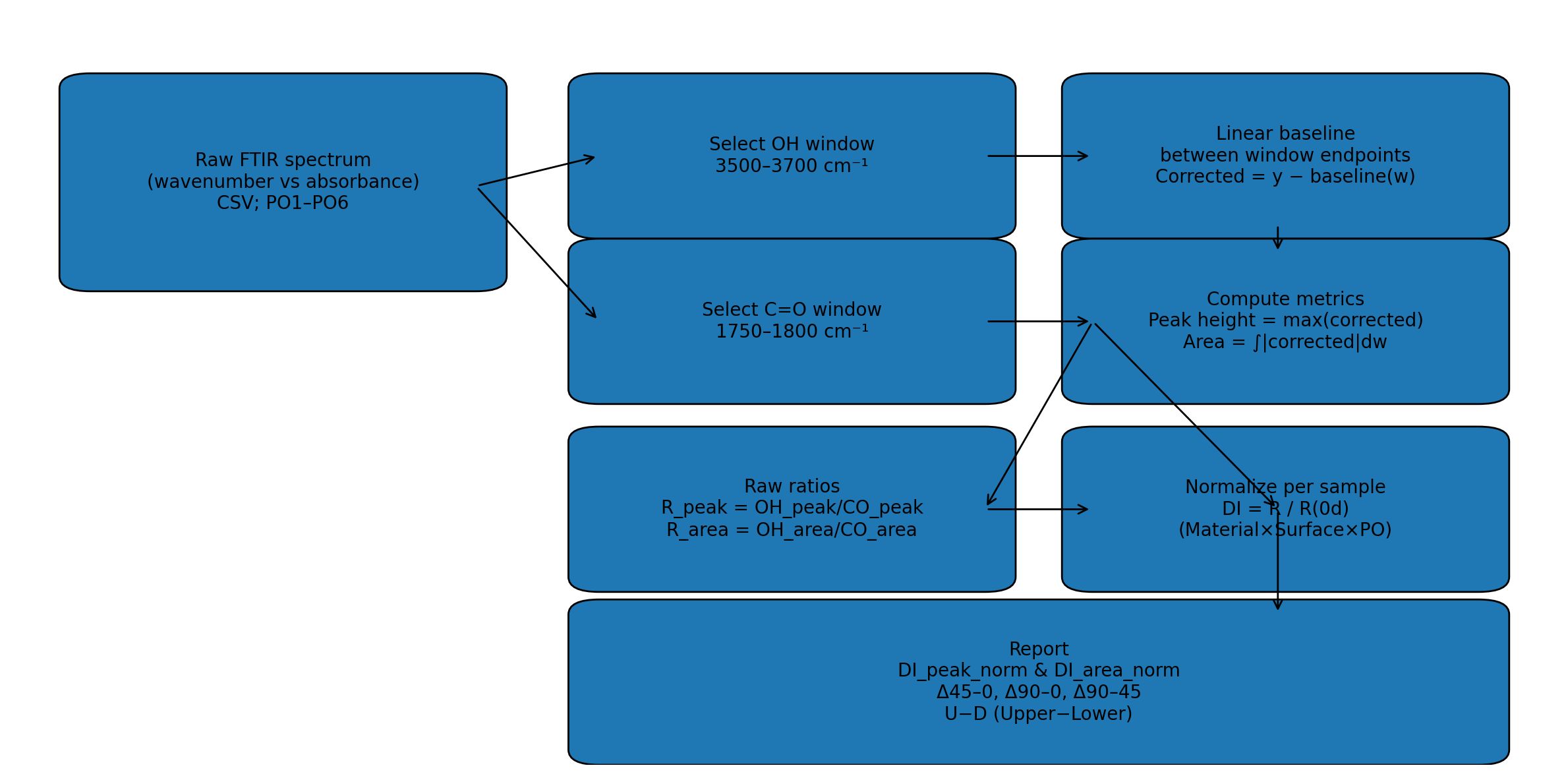

Supplement: Supplementary file 1 [file polymers-18-00963-s001.zip › polymers-4234996-supplementary/1- Figure S1 - FTIR_DI_Workflow_Diagram.jpg]
